# Comparing cross-sectional and longitudinal tracking to establish percentile data and assess performance progression in swimmers

**DOI:** 10.1038/s41598-022-13837-3

**Published:** 2022-06-18

**Authors:** Dennis-Peter Born, Eva Rüeger, C. Martyn Beaven, Michael Romann

**Affiliations:** 1 Swiss Swimming Federation, Section for High-Performance Sports, Bern, Switzerland; 2 Department for Elite Sport, Swiss Federal Institute of Sport Magglingen, Hauptstrasse 247, 2532 Magglingen, Switzerland; 3 University of Waikato, Te Huataki Waiora School of Health, Tauranga, New Zealand

**Keywords:** Quality of life, Health care

## Abstract

To provide percentile curves for short-course swimming events, including 5 swimming strokes, 6 race distances, and both sexes, as well as to compare differences in race times between cross-sectional analysis and longitudinal tracking, a total of 31,645,621 race times of male and female swimmers were analyzed. Two percentile datasets were established from individual swimmers’ annual best times and a two-way analysis of variance (ANOVA) was used to determine differences between cross-sectional analysis and longitudinal tracking. A software-based percentile calculator was provided to extract the exact percentile for a given race time. Longitudinal tracking reduced the number of annual best times that were included in the percentiles by 98.35% to 262,071 and showed faster mean race times (*P* < 0.05) compared to the cross-sectional analysis. This difference was found in the lower percentiles (1st to 20th) across all age categories (*P* < 0.05); however, in the upper percentiles (80th to 99th), longitudinal tracking showed faster race times during early and late junior age only (*P* < 0.05), after which race times approximated cross-sectional tracking. The percentile calculator provides quick and easy data access to facilitate practical application of percentiles in training or competition. Longitudinal tracking that accounts for drop-out may predict performance progression towards elite age, particularly for high-performance swimmers.

## Introduction

Previous studies have suggested longitudinal tracking of race times to assess talent development and performance trajectories^1^. Longitudinal tracking involves the retrospective analysis of performance of successful swimmers from age of peak performance (21–26 years of age) back to their adolescent performance^2^. As such, longitudinal tracking accounts for early drop-outs when predicting elite age success^3^. This is of particular importance, as success at junior age is only a poor predictor for success at elite age^4,5^. Of the successful swimmers under the age of 15 years, less than one third, i.e. 18 out of 60, were reselected for the senior team (aged ≥ 19 years)^4^. Furthermore, transition rate from junior to senior success was even as low as 10% for the top 10 male swimmers of the age category^5^. Although, transition rate improves with age (14 vs. 17 years of age)^5^, swimmers are typically selected at an early age^1,6^. Therefore, deselected talents are irreversibly lost at a timepoint with low transition rate. To account for these early drop-outs, longitudinal tracking appears to be the best choice when establishing reference values for talent development.

On the other hand, such longitudinal reference values may be biased by flaws in the current talent identification programs. As such, the relative age effect favors swimmers born early in the year^1^. At 13 years of age, 56% of Australian’s top national age-group swimmers were born in the first quarter of the year and only 4% in the last^1^. Additionally, at age of peak height velocity, i.e. 13.8 and 12.0 years for males and females, respectively, large inter-individual variation in maturation results in a biological age difference of up to 5 years^7,8^. Thus, early maturing swimmers are promoted for talent development programs, and potentially highly talented but late maturing swimmers can be deselected and irreversibly lost from the talent program^9^. The dominance of early maturing swimmers and those born early in the year may result in faster race times during adolescence^10^, especially in simultaneous strokes and sprint events^11^. Thus, expectations for talented, but normal or late maturing age-group swimmers would be overestimated. To reduce bias of current talent identification programs, previous studies analyzed race times using a cross-sectional approach and simply included all available data related to the research question^12^.

A recent study has established reference values based on percentile curves for Olympic swimming long-course events (50 m pool length)^3^. However, during the winter season in the northern hemisphere, races are held as short-course events. Competing in the same swimming strokes and over the same race distances, number of turns are increased due to the 25 m pool length. As such, at the beginning of each lap, swimmers achieve velocities far beyond the actual (clean) swimming velocity due to the push-off from the pool wall during each turn^13^. The different pacing pattern and velocity distributions improve short-course performance by 2.0 ± 0.6% for freestyle (FR) and 4.3 ± 3.2% for individual medley (IM) compared to the long-course events^14,15^. Therefore, specific reference values for short-course races are required, as they cannot be compared to long-course races.

Previous studies have analyzed performance development for a specific swimming stroke or race distance^5,16–18^. However, coaches and swimmers require reference values for both sexes, i.e. males and females, over all swimming strokes, i.e. butterfly (BU)–backstroke (BA)–breaststroke (BR)–FR–IM and all race distances, i.e. 50–100–200–400–800–1500 m. As such, race times across each age group (junior to elite age) differ for each of the aforementioned 34 swimming events and require specific reference values.

Modern technology enables us to gather the required data for both longitudinal and cross-sectional analyzes^19^. Specific percentile curves can be established for each swimming event, using the multiple million race results of the database of the European swimming federation (LEN)^20^. These percentiles provide a relative measure of race times enabling comparison between various race distances and swimming strokes^21^ across a wide range of performance levels and age groups^22^. However, to utilize the data in practice, coaches and other users need a software solution to avoid a time-consuming search of the required percentile values from multiple tables and charts.

Thus, the aims of the present study were to (1) establish reference values for competitive swimmers based on percentiles for swimming short-course (25 m pool length) races for both sexes over all swimming strokes and all race distances; (2) compare percentiles established by cross-sectional and longitudinal tracking; and (3) provide a software-based percentile calculator to enhance practical utility of the reference values for talent development.

## Results

**Figure 1:**
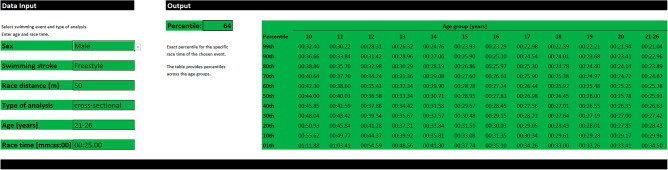
Screenshot of the percentile calculator that displays percentile data for a given race time of a particular swimming event. The software-based tool can be retrieved from the Supplementary Material.

Percentiles for the cross-sectional dataset were based on 15,928,723 annual best times. Longitudinal tracking reduced number of annual best times by 98.35% to 262,071 annual best times. Percentile data, including tables and figures for all 68 swimming events for both sexes, all swimming strokes, and all race distances, can be retrieved from the percentile calculator and tables in the Supplementary Material. Figure 1 illustrates the percentile calculator showing the exact percentile for a specific race time of the chosen swimming event.

Cross-sectional analysis resulted in slower mean race times compared to longitudinal tracking. This difference was significant in the early junior and late junior age category for upper (80–99th) percentiles (*P* < 0.05), in the early junior, late junior, and sub-elite age categories for medium (40–59th) percentiles (*P* < 0.05), and in all age categories for lower (1st–20th) percentiles (*P* < 0.05) for both male (Table 1) and female swimmers (Table 2). In summary, the lower the performance level, the more higher age categories were affected by the difference between cross-sectional analysis and longitudinal tracking.

**Table 1:** Comparison of percentiles based on cross-sectional vs. longitudinal analysis of *male* swimmers.

	Early junior *10–14 years*	Late junior *15–17 years*	Sub-elite *18–20 years*	Elite > *21 years*		*F*-value	*P*-value	_p_η^2^
**80–99th percentiles**								
Cross-sectional	02:34.31 ± 12.73	02:12.20 ± 10.36^#^	02:03.13 ± 09.02^#^	01:59.63 ± 08.86^#^	(a)	*F*_(1|198)_ = 10	*P* = 0.002	0.05
Longitudinal	02:27.70 ± 11.33*	02:06.04 ± 09.15*^#^	01:59.32 ± 08.24^#^	01:58.79 ± 08.24	(b)	*F*_(1|220)_ = 13,810	*P* < 0.001	0.99
					(c)	*F*_(1|220)_ = 114	*P* < 0.001	0.37
**40–59th percentiles**								
Cross-sectional	02:57.51 ± 12.99	02:28.91 ± 11.62^#^	02:18.07 ± 10.32^#^	02:16.83 ± 09.91^#^	(a)	*F*_(1|198)_ = 25	*P* < 0.001	0.11
Longitudinal	02:46.43 ± 11.88*	02:19.63 ± 09.98*^#^	02:11.70 ± 09.22*^#^	02:13.36 ± 09.37^#^	(b)	*F*_(1|220)_ = 19,143	*P* < 0.001	0.99
					(c)	*F*_(1|220)_ = 174	*P* < 0.001	0.47
**1st–20th percentiles**								
Cross-sectional	03:30.68 ± 18.08	02:54.75 ± 16.89^#^	02:42.92 ± 16.87^#^	02:46.26 ± 17.88^#^	(a)	*F*_(1|198)_ = 24	*P* < 0.001	0.11
Longitudinal	03:14.35 ± 16.36*	02:42.34 ± 15.12*^#^	02:34.51 ± 15.82*^#^	02:37.59 ± 16.00*^#^	(b)	*F*_(1|303)_ = 10,570	*P* < 0.001	0.98
					(c)	*F*_(1|303)_ = 90	*P* < 0.001	0.31

Analysis of variance (ANOVA) with repeated measure and one between subject factor was used to compare upper (80–99th), medium (40–59th), and lower percentiles (1st–20th) based on mean [mm:ss.00] ± standard deviation [ss.00] across the 200 m events of all swimming strokes.

(a) Main effect: type of analysis (cross-sectional vs. longitudinal).

(b) Main effect: age category (early junior−late junior–sub-elite–elite).

(c) Interaction effect: type of analysis × age group.

*Post-hoc* comparison.

*Significant difference to cross-sectional analysis.

^#^Significant difference to previous age category.

**Table 2:** Comparison of percentiles based on cross-sectional vs. longitudinal analysis of *female* swimmers.

	Age categories							
	Early junior 10–14 years	Late junior 15–17 years	Sub-elite 18–20 years	Elite > *21 years*		*F*-value	***P*-value**	_**p**_**η**^2^
**80–99th percentiles**								
Cross-sectional	02:38.57 ± 13.01	02:23.78 ± 11.50#	02:17.07 ± 10.66#	02:13.84 ± 10.69#	(a)	*F*_(1|198)_ = 11	*P* < 0.001	0.06
Longitudinal	02:29.53 ± 11.70*	02:16.71 ± 10.04*#	02:12.95 ± 09.59#	02:13.28 ± 09.71	(b)	*F*_(1|261)_ = 10,083	*P* < 0.001	0.98
					(c)	*F*_(1|261)_ = 394	*P* < 0.001	0.67
**40–59th percentiles**								
Cross-sectional	03:01.96 ± 13.11	02:42.76 ± 12.54#	02:35.01 ± 11.70#	02:35.45 ± 11.50	(a)	*F*_(1|198)_ = 28	*P* < 0.001	0.12
Longitudinal	02:48.28 ± 11.57*	02:32.53 ± 11.01*#	02:28.25 ± 10.47*#	02:31.62 ± 10.68#	(b)	*F*_(1|222)_ = 7111	*P* < 0.001	0.97
					(c)	*F*_(1|222)_ = 281	*P* < 0.001	0.59
**1st–20th percentiles**								
Cross-sectional	03:34.78 ± 17.78	03:11.49 ± 18.00#	03:05.15 ± 18.83#	03:11.06 ± 20.29#	(a)	*F*_(1|198)_ = 27	*P* < 0.001	0.12
Longitudinal	03:15.87 ± 16.10*	02:58.15 ± 16.84*#	02:55.09 ± 17.45*#	03:01.78 ± 18.03 *#	(b)	*F*_(2|305)_ = 2062	*P* < 0.001	0.91
					(c)	*F*_(2|305)_ = 80	*P* < 0.001	0.29

Analysis of variance (ANOVA) with repeated measure and one between subject factor was used to compare upper (80–99th), medium (40–59th), and lower percentiles (01–20th) based on mean [mm:ss.00] ± standard deviation [ss.00] across the 200 m events of all swimming strokes.

(a) Main effect: type of analysis (cross-sectional vs. longitudinal).

(b) Main effect: age category (early junior–late junior–sub-elite–elite).

(c) Interaction effect: type of analysis x age group.

*Post-hoc* comparison.

*Significant difference to cross-sectional analysis.

^#^Significant difference to previous age category.

Regarding cross-sectional analysis, race times significantly (*P* < 0.05) improved up to elite age for the high (80–99th) percentiles, compared to a plateauing in race times at sub-elite age when using longitudinal tracking. However, with both cross-sectional analysis and longitudinal tracking for male and female swimmers, race times of the low (1st–20th) percentiles became significantly faster (*P* < 0.05) up to sub-elite age but significantly slower again at elite age.

## Discussion

The present study provides percentile curves for competitive swimmers for short-course race times from 10 years of age to age of peak performance (21–26 years) for both sexes, all swimming strokes, and all race distances. Longitudinal tracking reduced the number of subjects included, and resulted in faster race times compared to the cross-sectional analysis. The older the age category, the more similar became cross-sectional to longitudinal data for the high but not low percentiles. Regarding low (1st–20th) percentiles, race times became faster up to sub-elite age, before deteriorating at elite age both with cross-sectional and longitudinal tracking. High percentile (80–99th) race times improved up to elite age when cross-sectionally analyzed but plateaued at sub-elite age when using longitudinal tracking. A software-based percentile calculator was provided that enables coaches and performance analysts to determine the exact percentile for a particular race time and age group for each of 34 swimming events.

Successful elite age swimmers outperform their lower ranked peers from the age of 12 years onwards^17^. Therefore, longitudinal tracking, i.e. swimmers that were retrospectively tracked from peak performance age, showed faster race times during adolescence compared to cross-sectional data, which included all swimmers in each age-group regardless of later drop-outs. Interestingly, regarding high percentiles, cross-sectional data aligned with longitudinal data at sub-elite age. Due to the low transition rate from junior to senior age^4,5^, swimmers are less likely to drop-out once they reach sub-elite age category^5,23^. Additionally, performing within the high percentile range requires a structured training process and performance-oriented attitude^24^. Swimmers competing for fun rather than success likely either drop from the performance level (80–99th percentiles) or out of the sport, while other swimmers accumulate valuable training time and competition experience on their way to elite age success^25,26^. This intended or natural deselection may explain the lack of a performance difference between cross-sectional and longitudinal data at the high percentiles after sub-elite age.

It is clear that longitudinal tracking dramatically reduced number of subjects included compared to the cross-sectional analysis. Longitudinal tracking excludes data from poor performers who compete for reasons other than winning and eventually drop out of competitive swimming during or after junior age^23^. Therefore, percentiles established on retrospective analysis from peak performance age are appropriate to predict performance progression towards elite age^1^. This improves practical utility of the percentiles, in particular for the high percentile range, and provides “realistic data on the long-term potential”^5^ to establish development guidelines for high-performance swimmers.

Relative age and biological maturation are known to affect swimming performance during adolescence^27,28^ and may therefore bias longitudinal percentile data. For instance, the same race time, e.g. 02:22.00 [mm:ss.00] in 200 m FR, is rated on the 90th or 68th percentile for a 12 or 13 year old male swimmer, respectively. Still, early (January 1st) and late (December 31st) in the year born swimmers, which have a one-year age difference, are assessed within the same age-group. Therefore, previous studies have developed corrective adjustments for the relative age effect in swimming, which should be considered when interpreting percentiles^1,29^. However, biological age could not be determined within the present study due to 31.6 million race data included. From a practical perspective, coaches can determine the maturity off-set using invasive or non-invasive methods^7,8,30^ and thus, use the biological, as opposed to the chronological, age of their swimmers for the present percentiles and calculator software. Additionally, bio-banding, i.e. grouping junior athletes for competitions based on biological rather than chronological age^31^, reduced physical but increased technical demand of soccer match play^32^. Bio-banding has not yet been scientifically evaluated in swimming. However, with the reduced physical advantage of early maturing individuals^31,32^, bio-banding may improve technical development of early and selection chances for late maturing swimmers.

Irrespectively of chronological and biological age differences, the present percentiles were established to assess progression of swimming performance. The aim was to add a variable to talent identification beyond the traditional and one-dimensional comparison of current race times with swimmers from the same age-group. As such, during adolescence, swimmers’ race times continuously improve due to growth^1,18^. Swimmers with average development are expected to follow a particular percentile over the years. However, a lower percentile ranking from one year to another would indicate underperformance despite faster race times. In contrast, some late maturing swimmers may show medium percentile race times. However, annually improved percentile rankings would indicate effective training and high trainability, e.g. the genetic capacity to adapt to training^6,33,34^. As such, a swimmer’s overall trajectory may still be worthwhile to achieve a high percentile when reaching elite age with a possible performance progression of 34–42% from 8 to 18 years of age^18^. Therefore, assessment of trainability, along with tight monitoring of performance progression, i.e. percentile ranking and its annual development, may help to discover potentially overlooked talents, even without knowledge of the maturation status. Still, coaches and federation officials should be aware of the relative age effect, which is larger at younger ages than covered by the present percentiles (< 10 years), and incorporate corrective adjustments^1,28^.

Finally, percentiles may help identify the event in which junior swimmers have their largest potential. Junior swimmers typically compete in multiple swimming strokes and race distances for a broad technical and physiological education^35,36^, and the individual’s strongest events are typically selected based on success in regional and national championships. However, success is affected by performance of the other competitors and the overall performance level of the region and nation. Thus, comparing percentile rankings and their annual development between various swimming strokes and race distances may help identify a swimmer’s strengths and weaknesses, as the present percentiles normalize race times to a standard score and provide a relative measure of swimming performance based on international race data^21,37^.

In conclusion, the present study provides percentiles curves for competitive swimmers for all swimming strokes, all race distances, and both sexes for short-course pool events. Longitudinal tracking showed significantly faster race times compared to cross-sectional data. In particularly for the high percentiles, i.e. 80–99th, and high-performance swimmers, for drop-outs accounted longitudinal tracking may predict progression towards elite age. The percentile calculator facilitates quick and easy data access for practical application of percentiles in training and competition, while avoiding the inconvenience of searching the exact percentile for a given race time and age from 68 charts or tables with up to 1188 data points each. Performance analysts and coaches can use the percentiles to assess race times and establish individual performance trajectories and define realistic goals for young talented swimmers. As swimmers with average development are expected to follow a particular percentile over the years, the combination of two factors, i.e. current percentile ranking and changes over time, can help to assess trainability and identify talented swimmers during junior age.

## Methods

### Sample

Race times were provided by the publicly available database of the European Swimming Federation LEN (Ligue Européenne de Natation)^20^. A total of 31,645,621 short-course (25 m pool length) race times from 2003 to 2019 were included in the study. The study was pre-approved by the internal review board of the Swiss Federal Institute of Sport Magglingen (Reg.-Nr. 124_LSP_201221_234-3.2.127) and is in accordance with the ethical charta of the World Health Organization for studies on human subjects (Helsinki Declaration). No informed consent of the subjects was required, as race times and age were retrieved from a publicly available database.

### Data collection/analysis

Two datasets of percentiles were established. The first dataset involved a cross-sectional analysis based on individual annual best times of all swimmers from the database in that period of time (2003–2019) for each specific swimming event. The second dataset involved longitudinal tracking to account for drop-out during junior age. As such, swimmers (n = 8205) were only included if they still competed at age of peak performance, which occurs between 21 and 26 years of age^2^. For each specific swimming event, swimmers at peak performance age were identified in the 2019 dataset and their annual best times were tracked retrospectively. A minimum of two individual annual best times (one in 2019 and one in another year of the time period investigated) in the particular swimming event were required to be included in the longitudinal analysis.

Percentiles were established for both male and female swimmers across all swimming strokes and all race distances, i.e. BU (50–100–200 m), BA (50–100–200 m), BR (50–100–200 m), FR (50–100–200–400–800–1500 m), and IM (200–400 m). Swimmers typically start their talent pathway with learn-to-swim programs aged 6–10 years^38^. Subsequently, talented swimmers transition to competitive sports with participation in regional and national competitions. During the initial years of the talent pathway, race distances typically increase with age^39,40^. Therefore, percentiles were established from the age of 10, 11, 12, and 13 years of age for 50 m, 100 m, 200 m/400 m, 800 m/1500 m events, respectively. To compare the cross-sectional and longitudinal approach, mean race times were compared for each age category: early junior age (10–14 years), late junior age (15–17 years), sub-elite age (18–20 years), and elite age (21–26 years).

Annual best times that were slower than three times the standard deviation for a particular age group were excluded as outliers^41^. Following the exclusion of 968,770 outliers (3.06%) from the dataset, percentiles were calculated, with the z-score around the median^37^. The Lamda-Mu-Sigma (LMS) method was applied to normalize data and account for potential right- and left-sided skewness^37,42^. The LMS method corrects skewness (L) with the median (M) and coefficient of variation (S), so that the z-score is a valid indicator for the percentiles^37^. The LMS method is particularly useful when describing non-linear percentile curves during growth and adolescence^22,37,43^. For the diagrams, percentile curves were smoothed using the cubic spline interpolation^37^. Percentiles and diagrams were established with RStudio (version 1.1.456, RStudio Team, Boston, United States).

### Percentile calculator

A software-based percentile calculator was provided to allow easy access to the percentiles based on the large dataset, which includes 34 swimming pool events. Based on the = VLOOKUP function in Microsoft Excel (Microsoft Corporation, Redmond, WA), the percentile for a particular race time is displayed based on the chosen sex, swimming stroke, and race distance. Additionally, a table of the selected swimming event is displayed to provide an overview of the percentiles across all age categories.

### Statistical analysis

A two-way analysis of variance (2 × 4 ANOVA) with repeated measures and one between-subject factor: type of analysis (cross-sectional vs. longitudinal) × age category (early junior − late junior − sub-elite − elite) was used to compare mean values ± standard deviation of the upper (80th–99th), medium (40th–59th), and lower percentiles (1st–20th) with a Tukey’s *post-hoc* test, where partial eta-squares indicate a small (0.01), medium (0.06), and large (0.14) effect^44^. If variances were not equal based on the Levene’s test, a Bonferroni’s *post-hoc* test was applied^41^. The Greenhouse–Geisser correction was applied to the main effects with unequal variances of the within-subject factors based on an ε < 0.75 in Mauchly’s test of sphericity^41^. As the 200 m events are the only common race distances across all swimming strokes for Olympic swimming events^45^, the 200 m race times across all swimming strokes were used for the statistical analysis. Normality was confirmed by Gaussian distribution in the histogram and standardized residuals showing a diagonal straight line in the Q–Q plot^41^. An alpha-level of 0.05 indicated statistical significance. Statistical analyses were performed using the JASP statistical software package version 0.14 (JASP-Team, University of Amsterdam, Amsterdam, The Netherlands).

## Supplementary Information

Supplementary Information 1.

Supplementary Information 2.

## Data Availability

All data generated and analyzed during the study are included in the published article and its supplementary information file.

## Abbreviations

BU: Butterfly
BA: Backstroke
BR: Breaststroke
FR: Freestyle
IM: Individual medley
