# Supplementary material for: Comparing cross-sectional and longitudinal tracking to establish percentile data and assess performance progression in swimmers
Source: Sci Rep. 2022 Jun 18;12:10292. doi: 10.1038/s41598-022-13837-3 (PMC9206680; doi:10.1038/s41598-022-13837-3)

Male swimmers

Butterfly (BU)

M_BU_50

Cross-sectional analysis

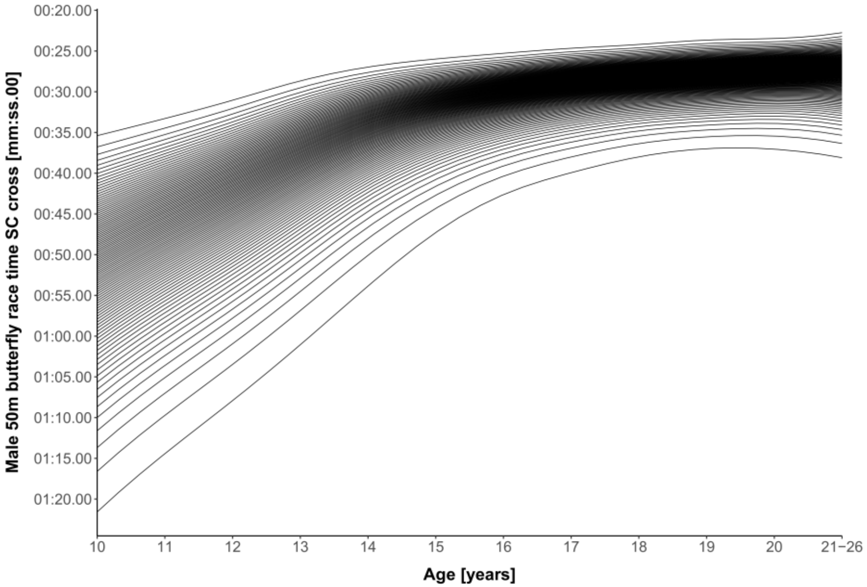

Longitudinal tracking

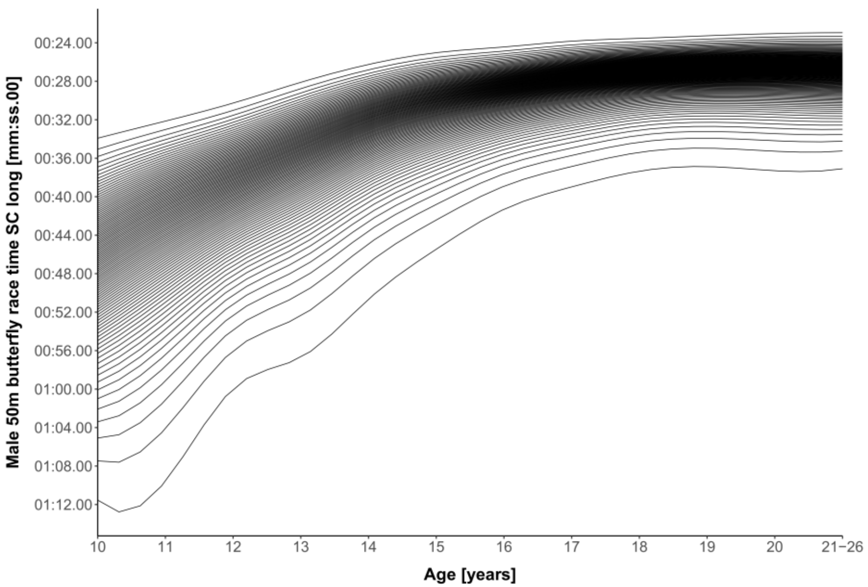

M_BU_100

Cross-sectional analysis

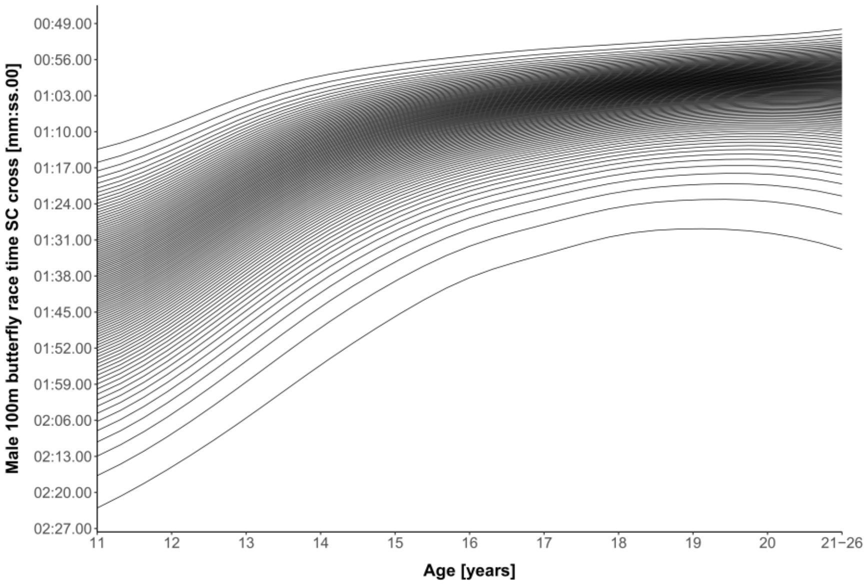

Longitudinal tracking

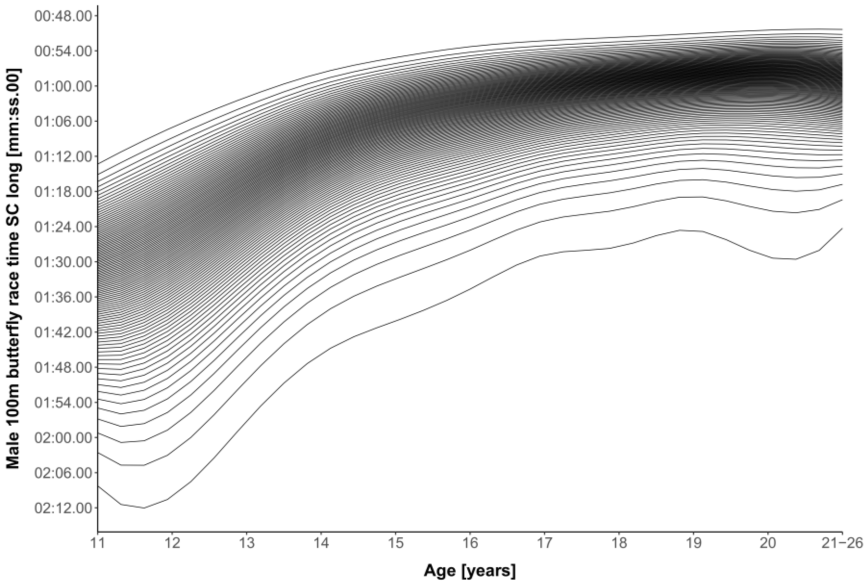

M_BU_200

Cross-sectional analysis

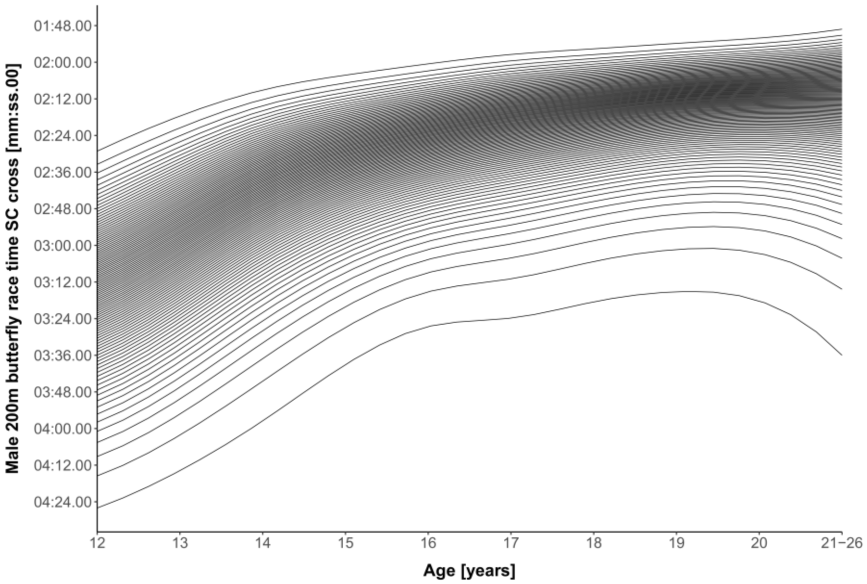

Longitudinal tracking

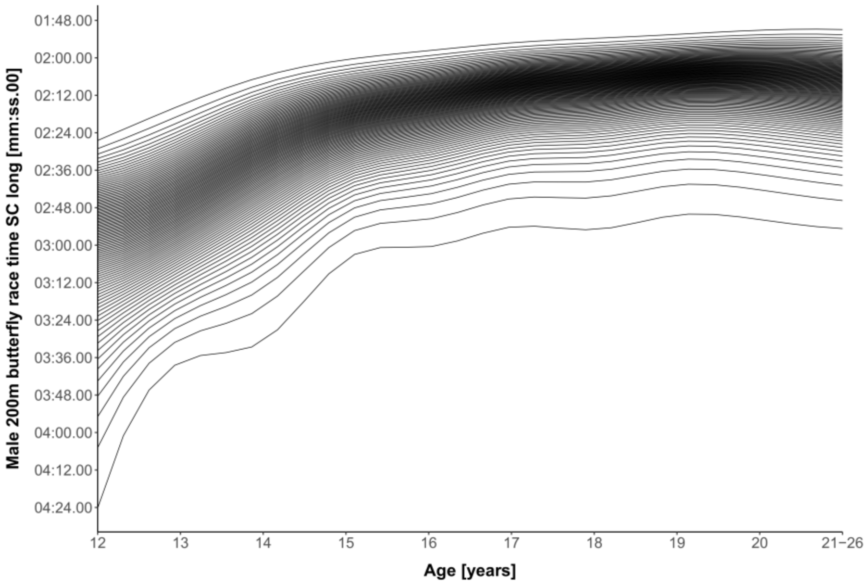

Backstroke (BA)

M_BA_50

Cross-sectional analysis	Longitudinal tracking
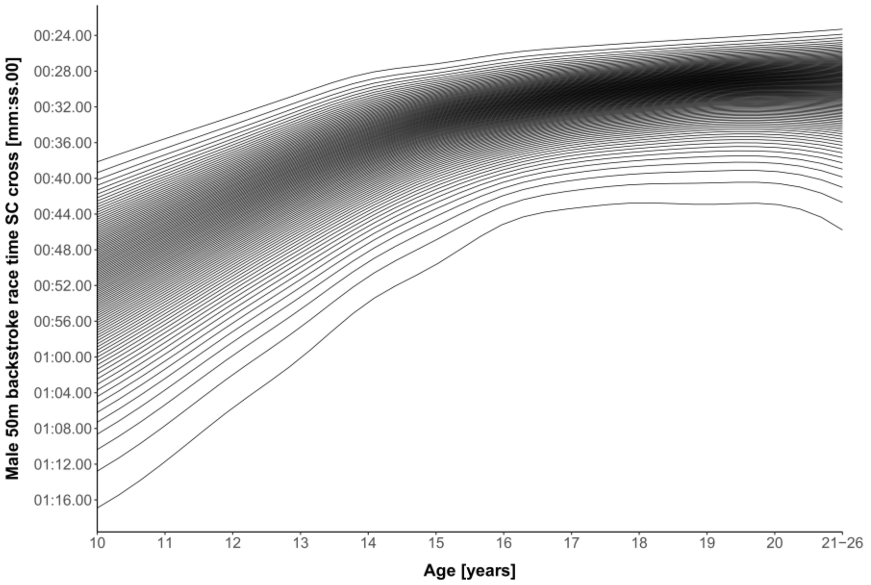	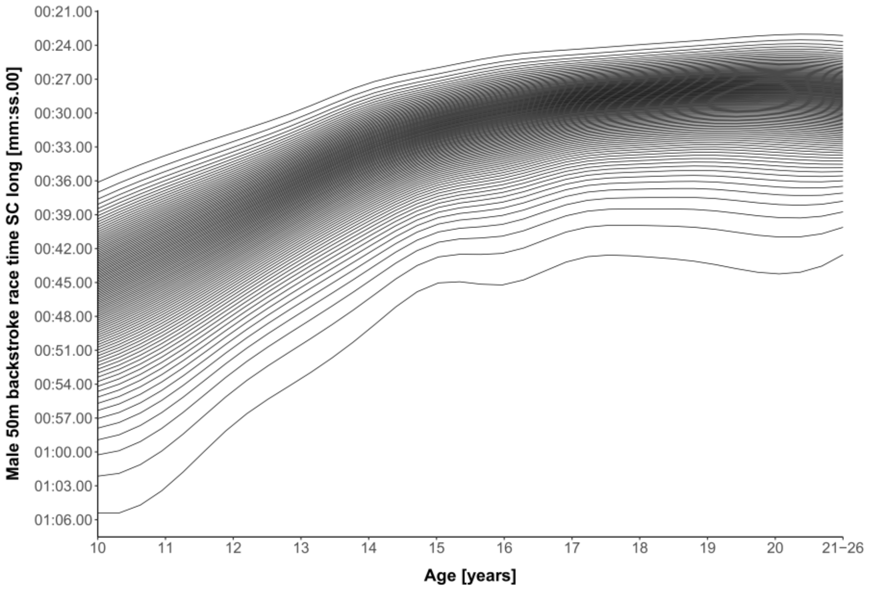

M_BA_100

Cross-sectional analysis

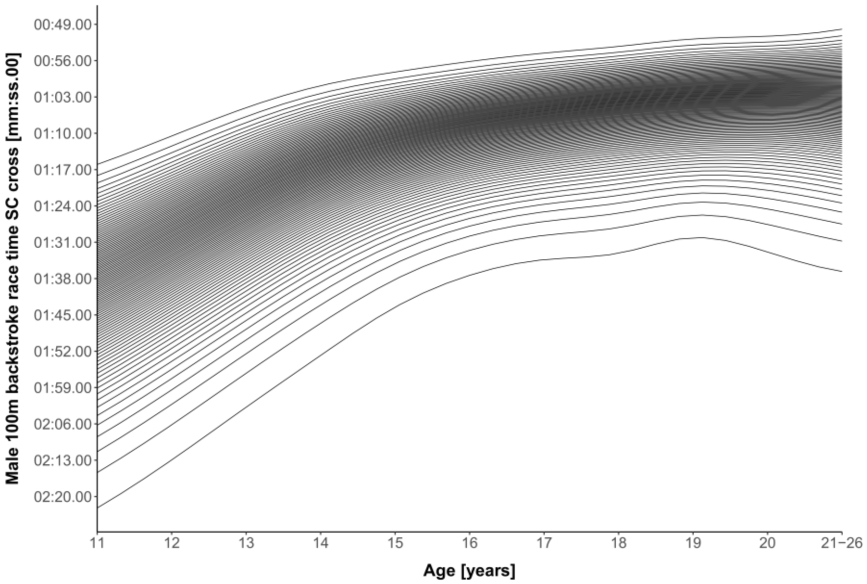

Longitudinal tracking

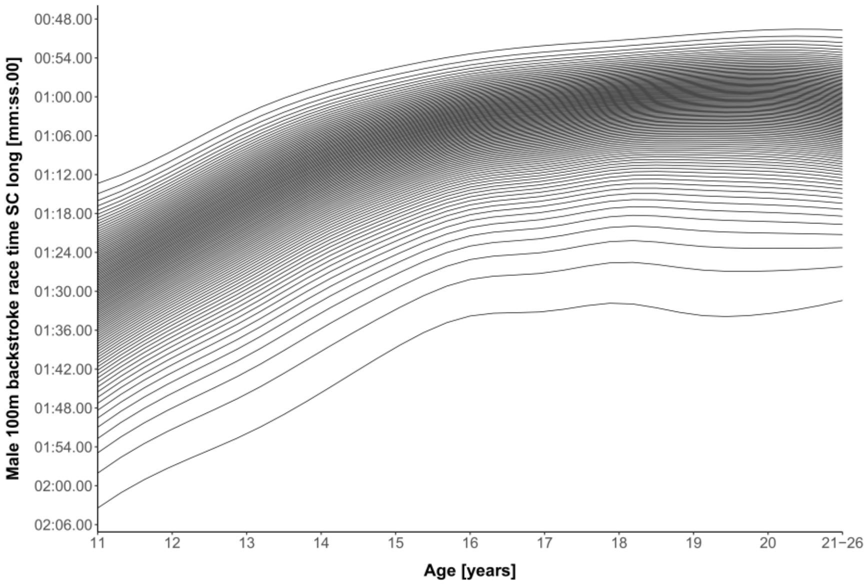

M_BA_200

Cross-sectional analysis

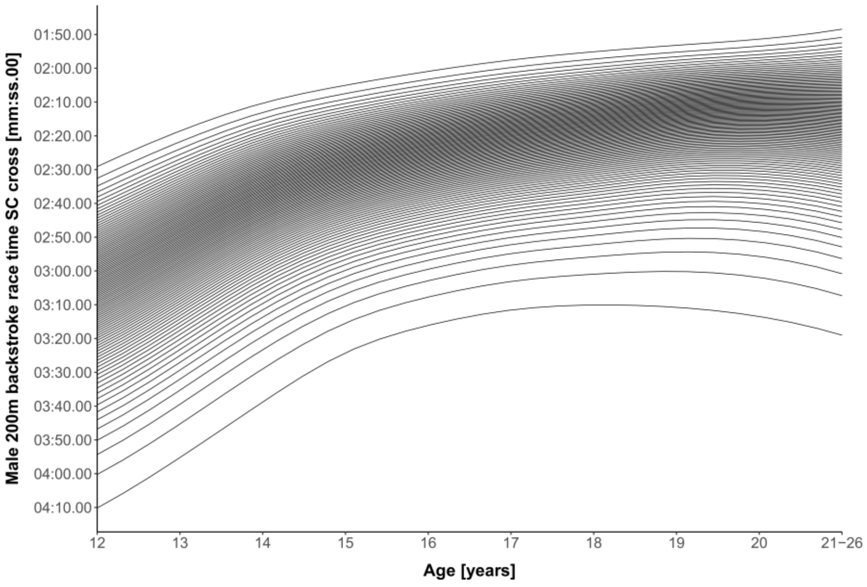

Longitudinal tracking

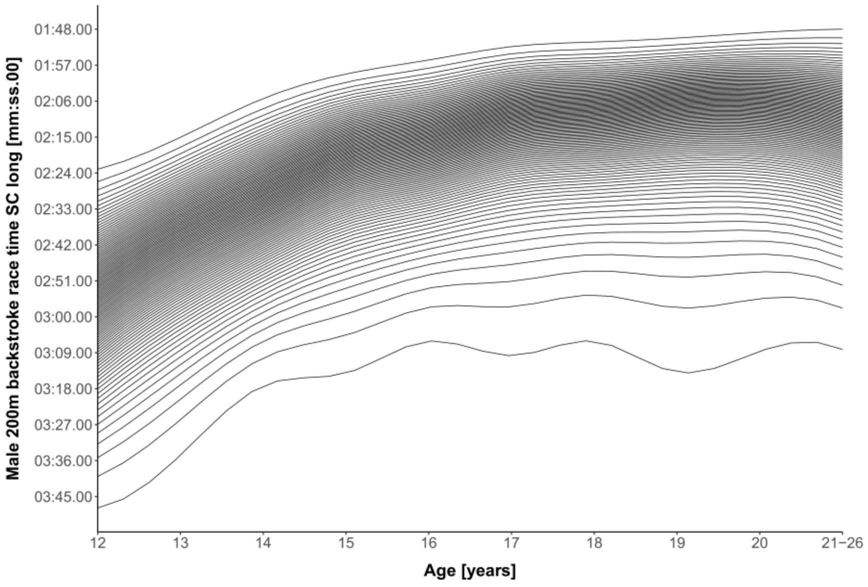

Breaststroke (BR)

M_BR_50

Cross-sectional analysis

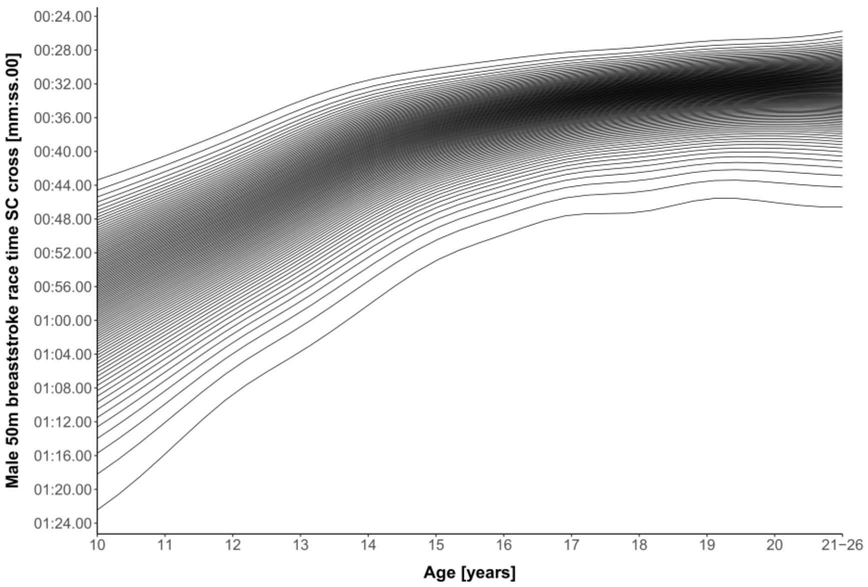

Longitudinal tracking

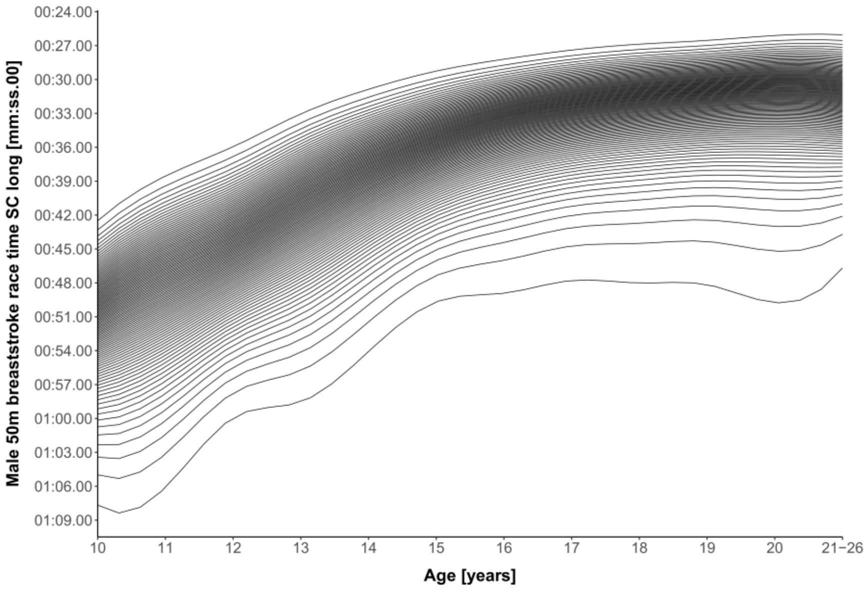

M_BR_100

Cross-sectional analysis	Longitudinal tracking
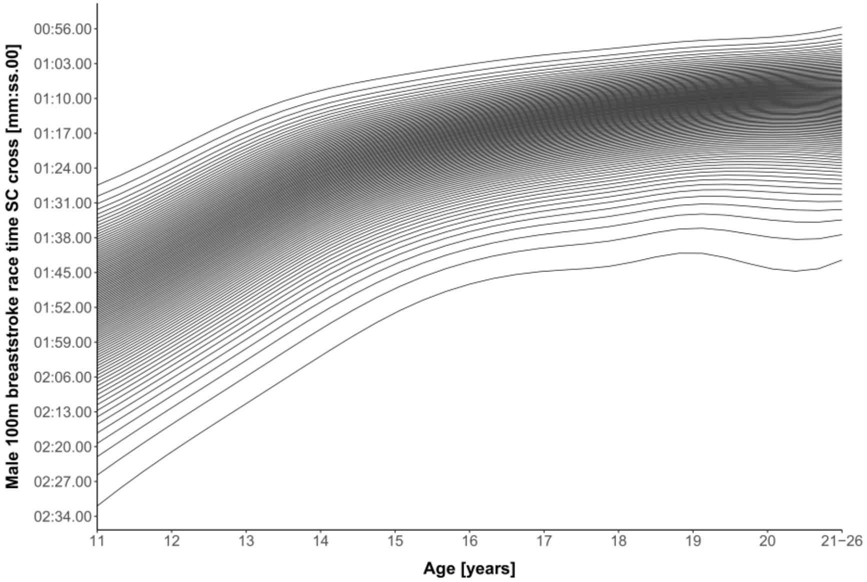	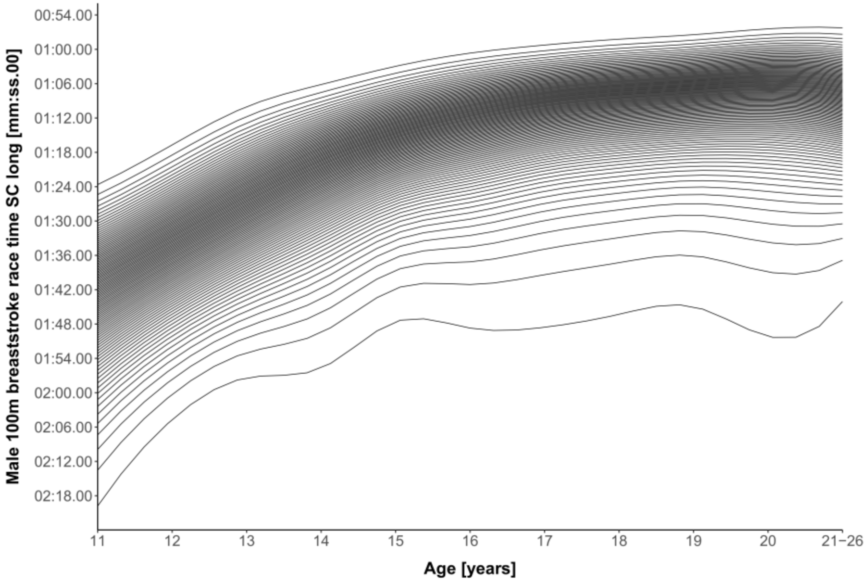

M_BR_200

Cross-sectional analysis

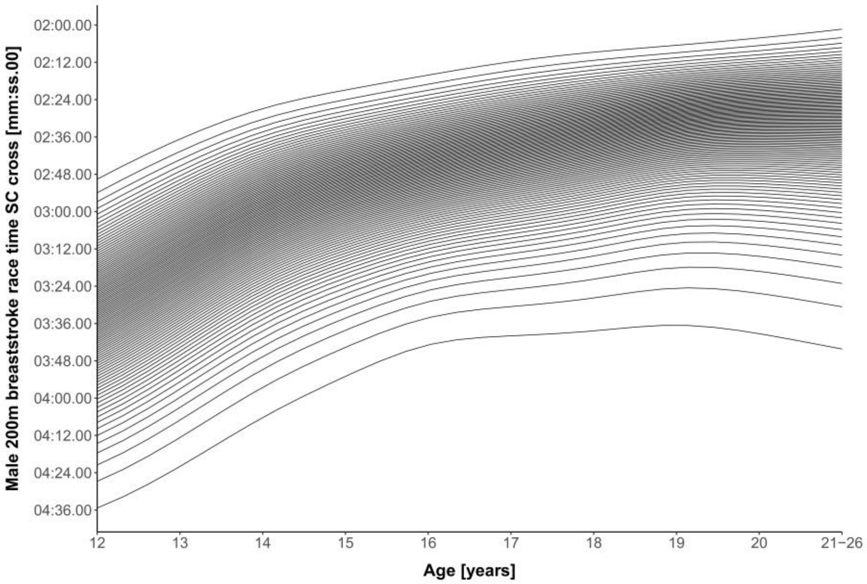

Longitudinal tracking

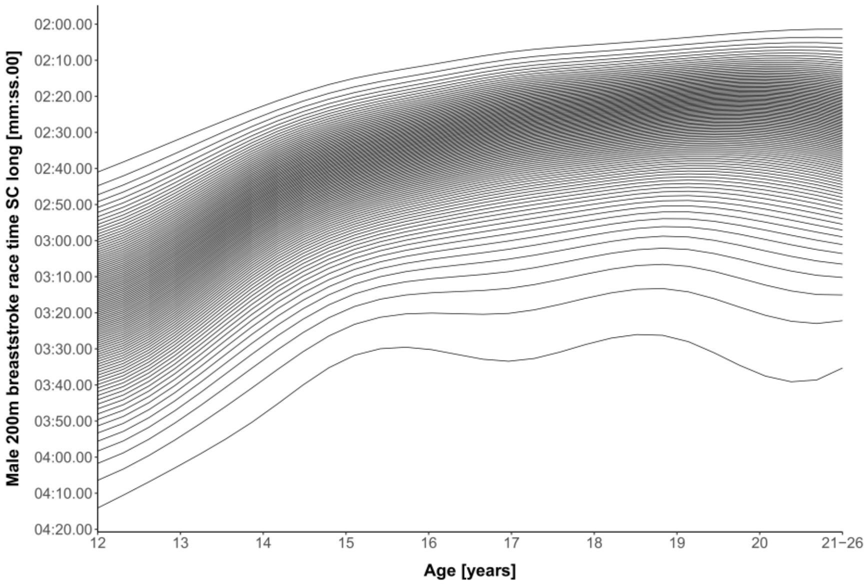

Freestyle (FR)

M_FR_50

Cross-sectional analysis	Longitudinal tracking
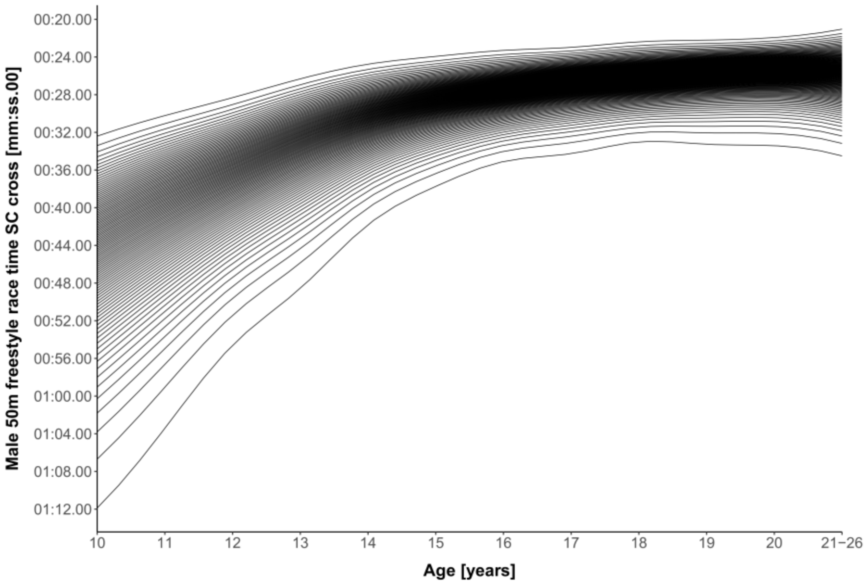	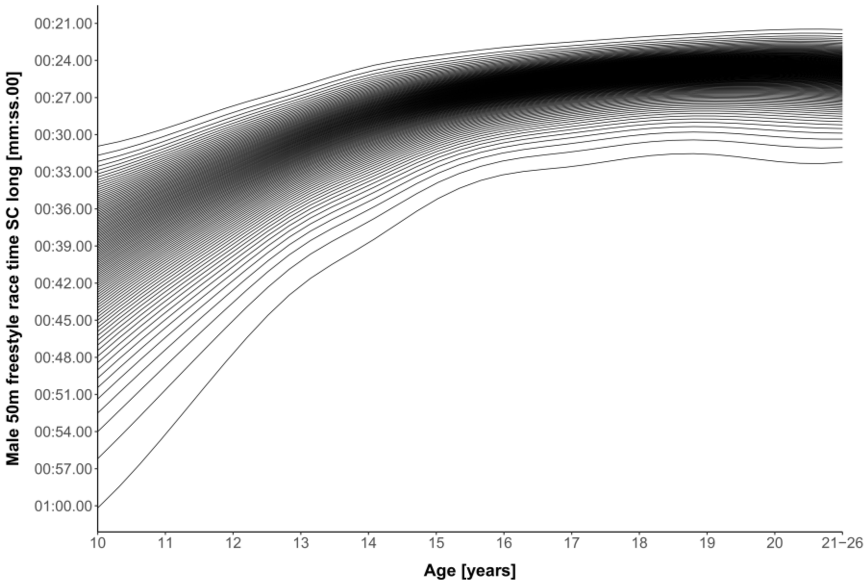

M_FR_100

Cross-sectional analysis

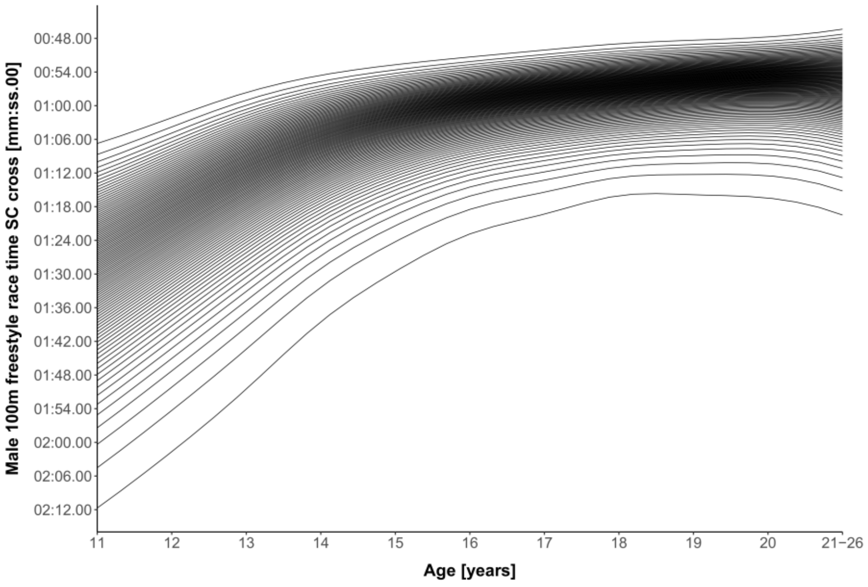

Longitudinal tracking

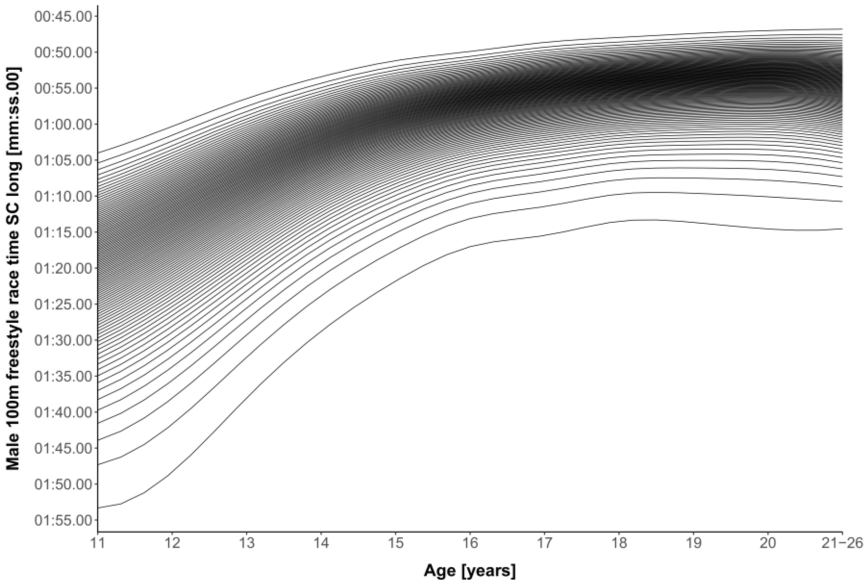

M_FR_200

Cross-sectional analysis	Longitudinal tracking
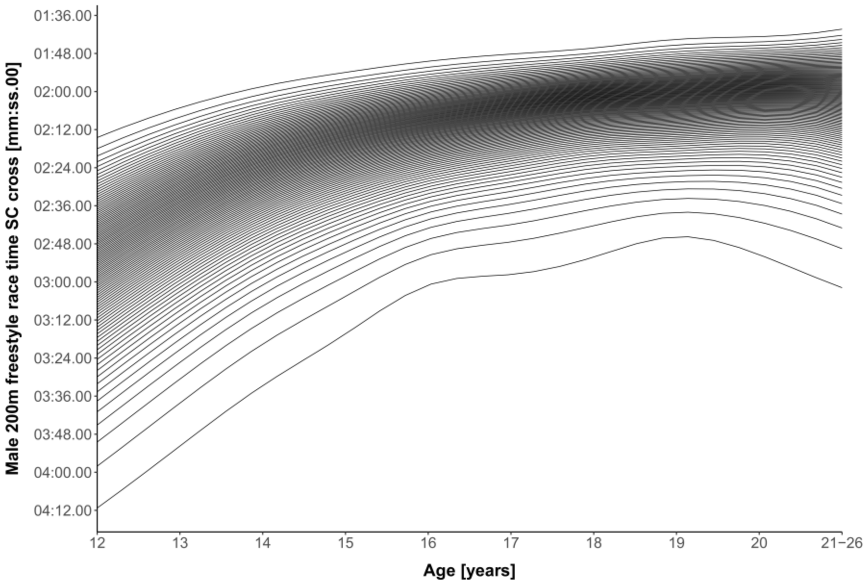	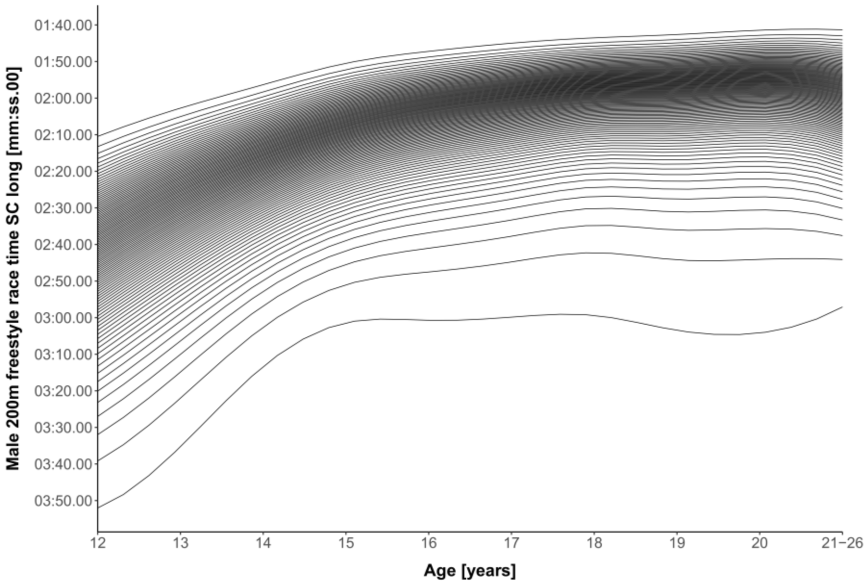

M_FR_400

Cross-sectional analysis

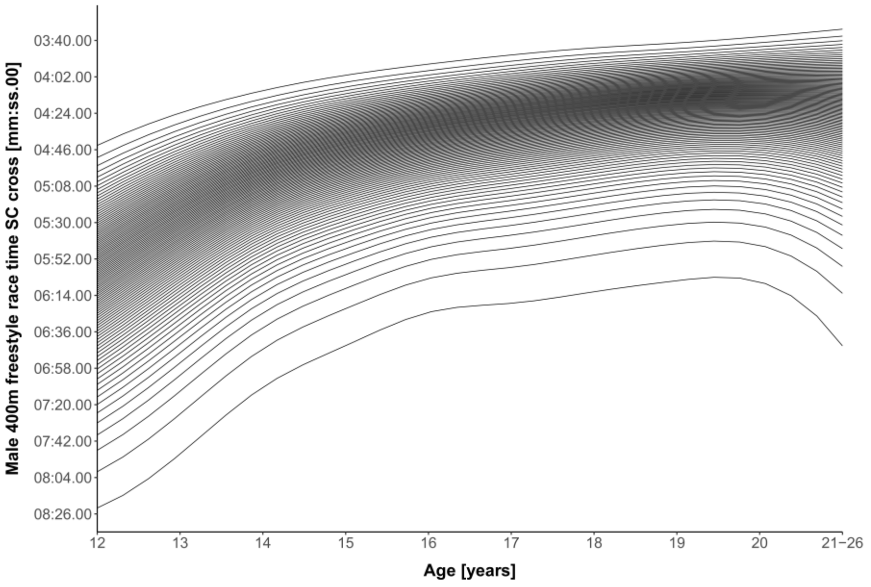

Longitudinal tracking

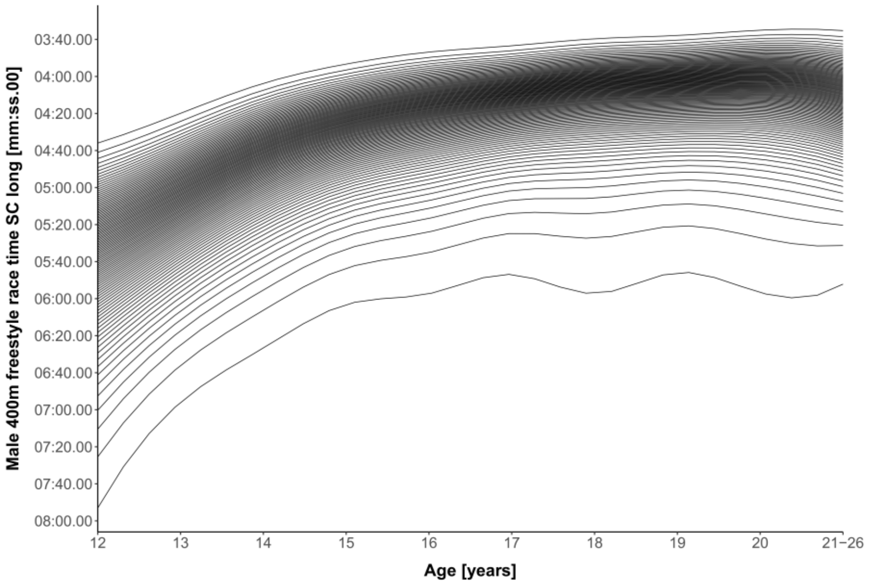

M_FR_800

Cross-sectional analysis

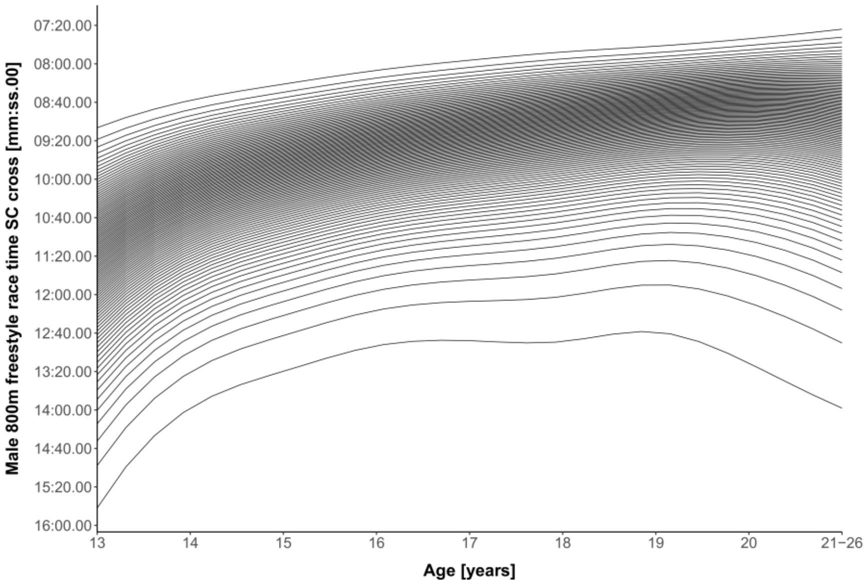

Longitudinal tracking

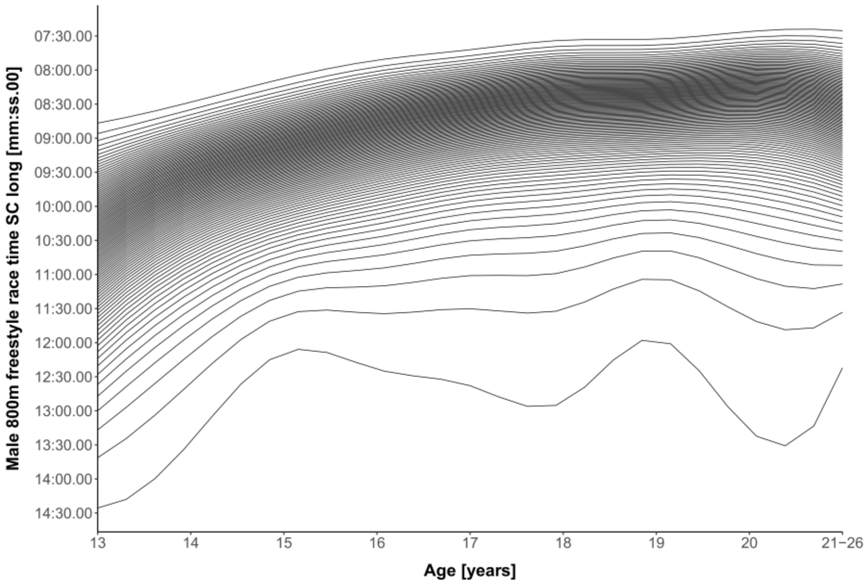

M_FR_1500

Cross-sectional analysis	Longitudinal tracking
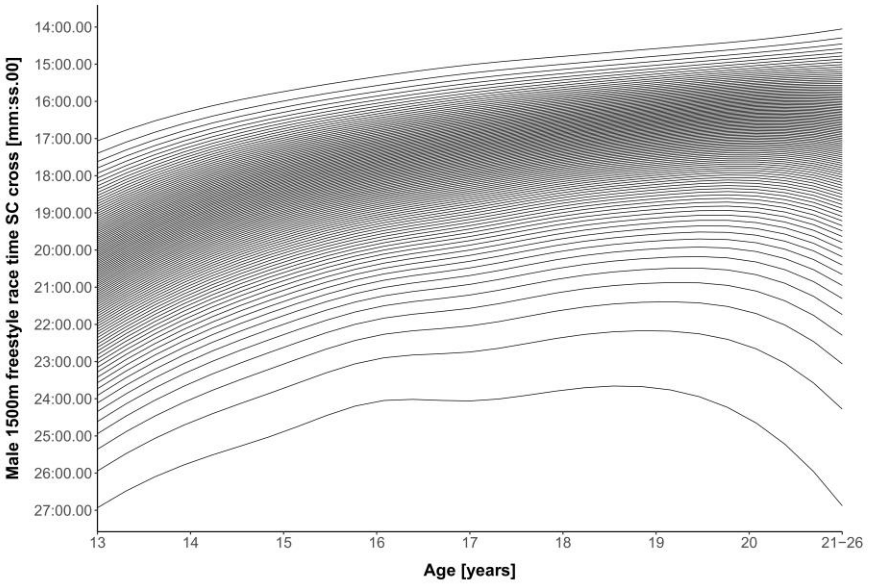	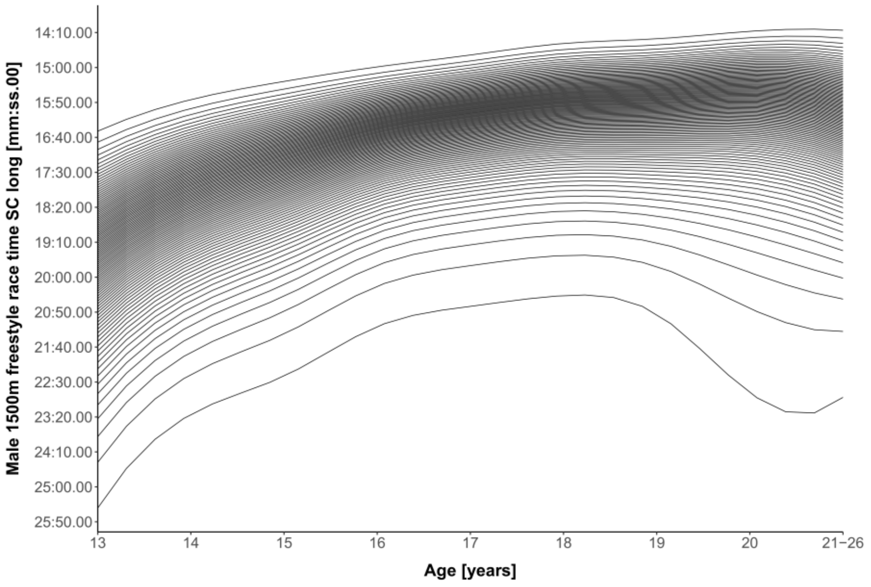

Individual medley (IM)

M_IM_200

Cross-sectional analysis

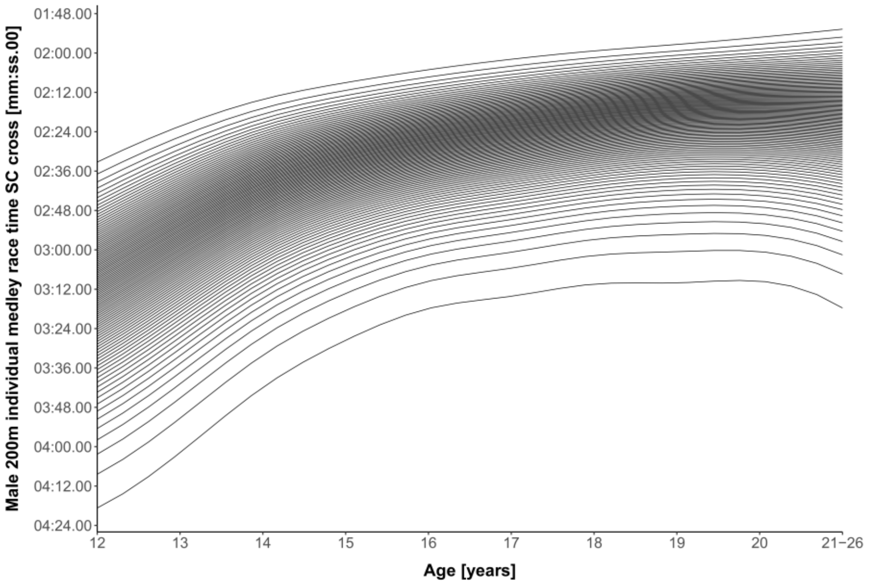

Longitudinal tracking

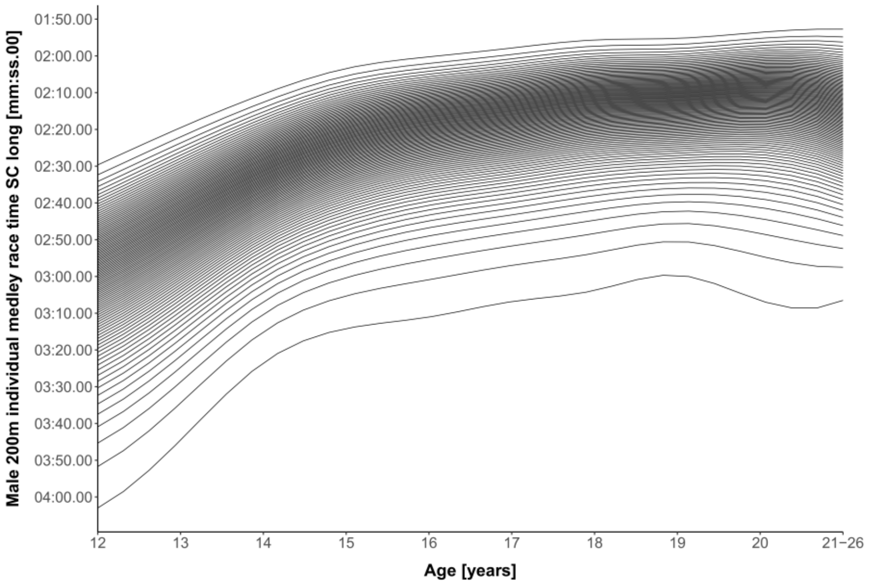

M_IM_400

Cross-sectional analysis	Longitudinal tracking
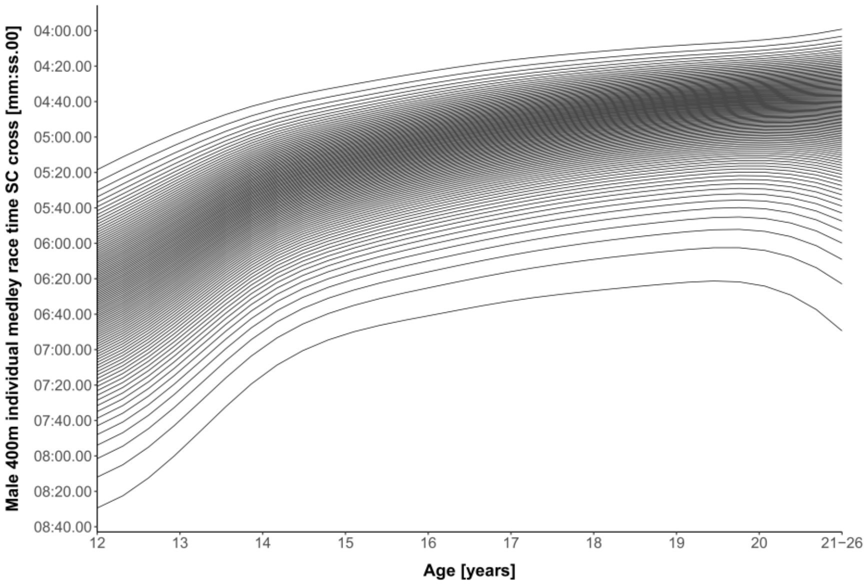	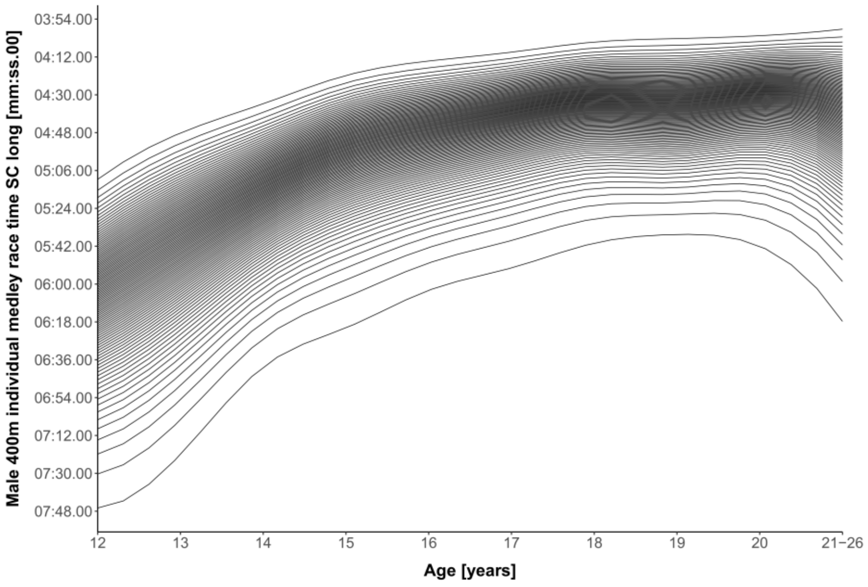

Female swimmers

Butterfly (BU)

F_BU_50

Cross-sectional analysis	Longitudinal tracking
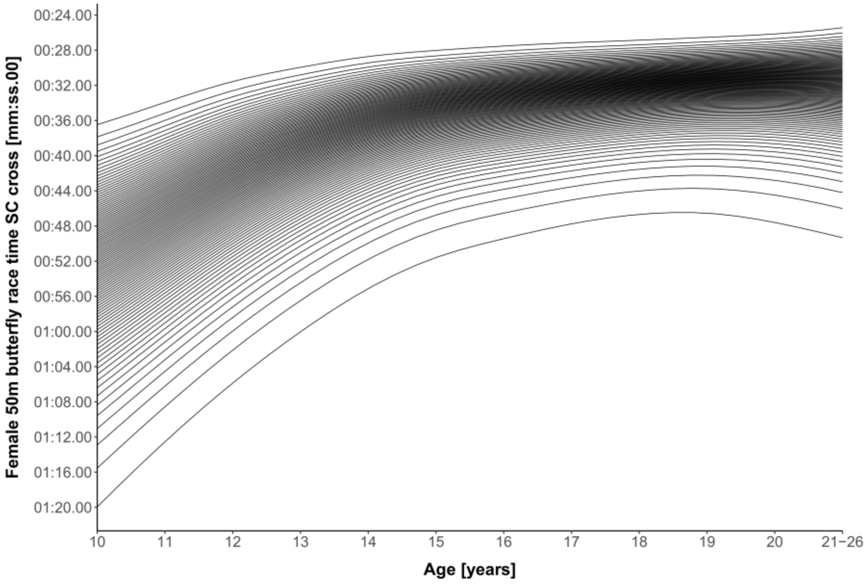	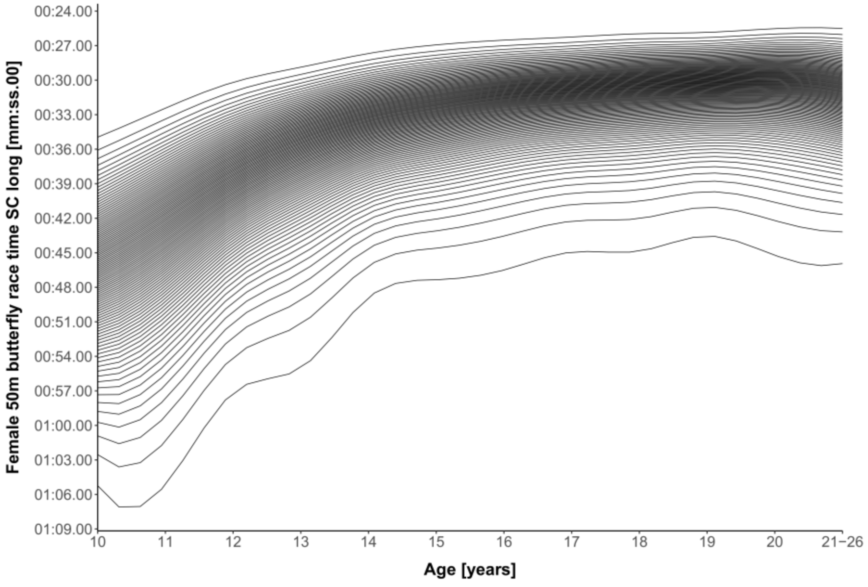

F_BU_100

Cross-sectional analysis	Longitudinal tracking
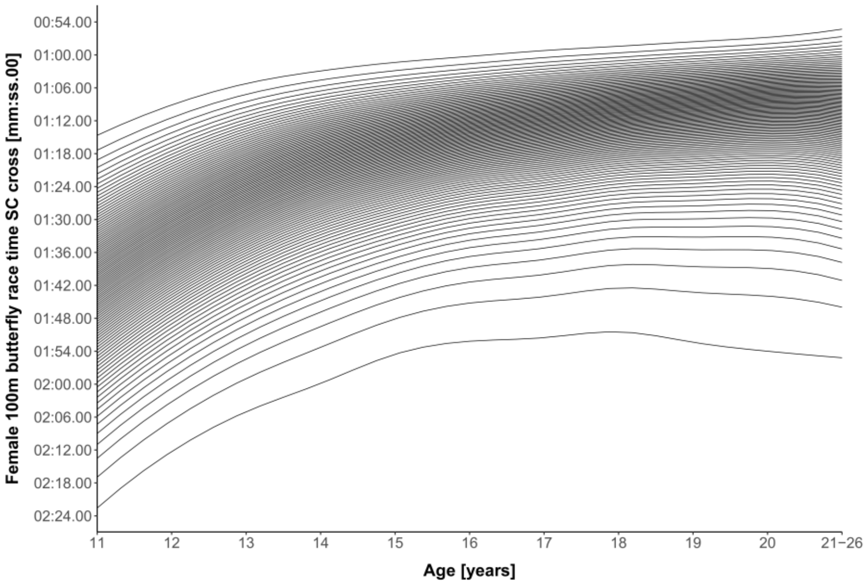	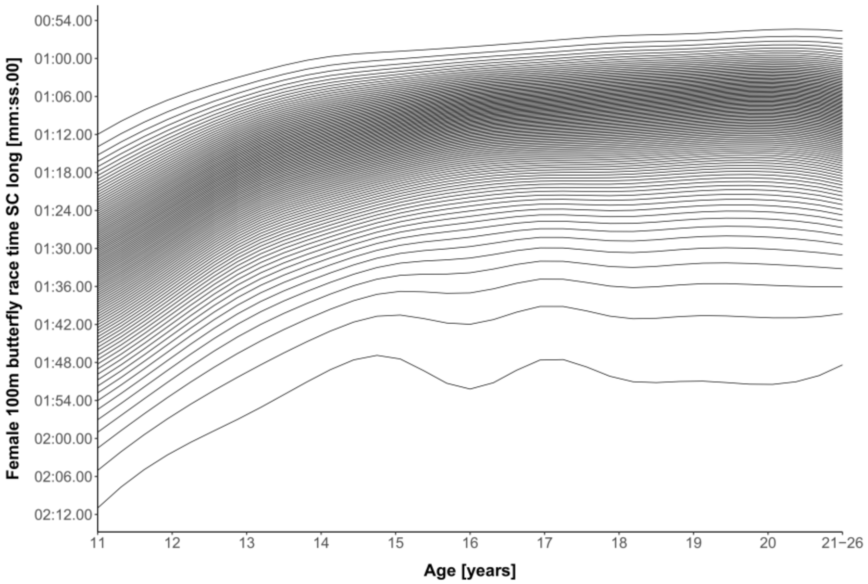

F_BU_200

Cross-sectional analysis

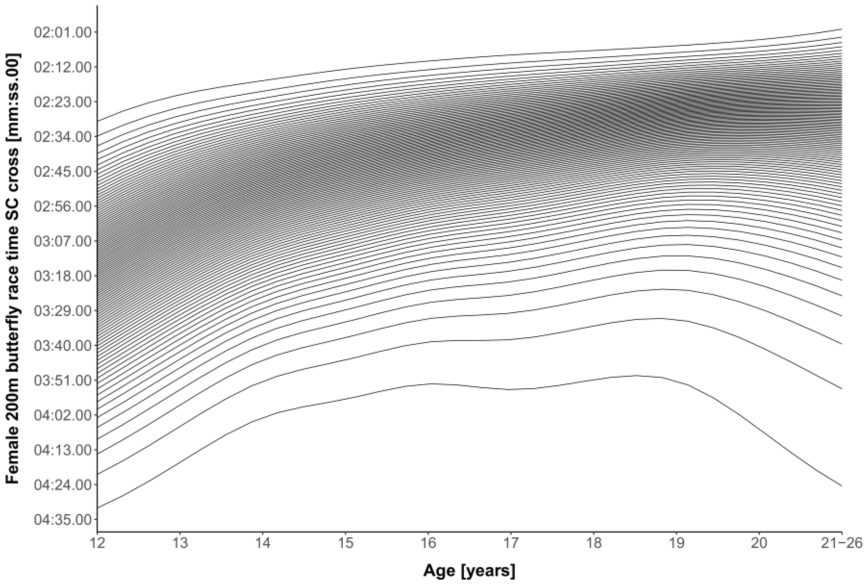

Longitudinal tracking

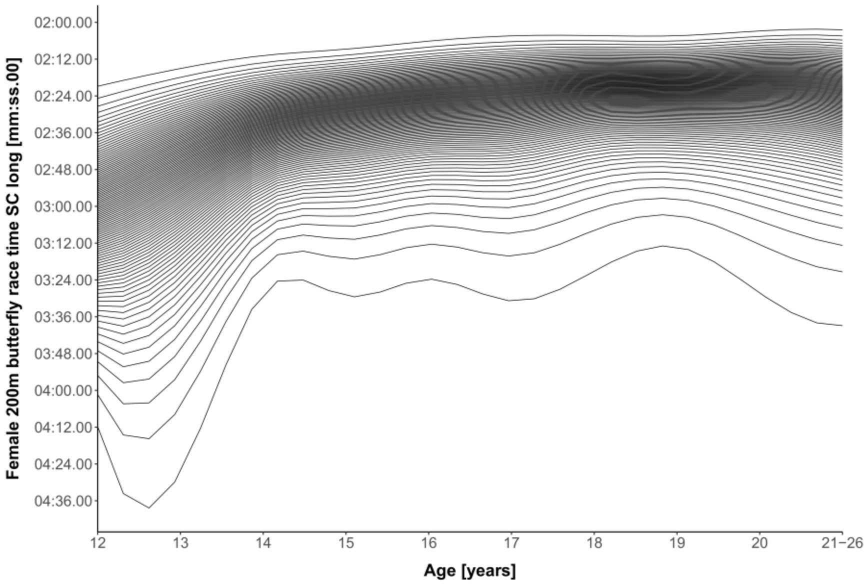

Backstroke (BA)

F_BA_50

Cross-sectional analysis

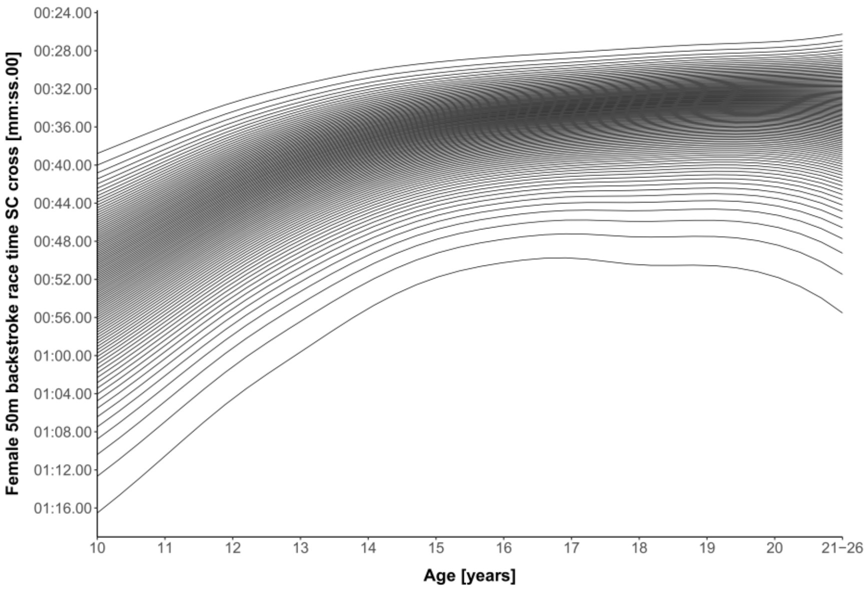

Longitudinal tracking

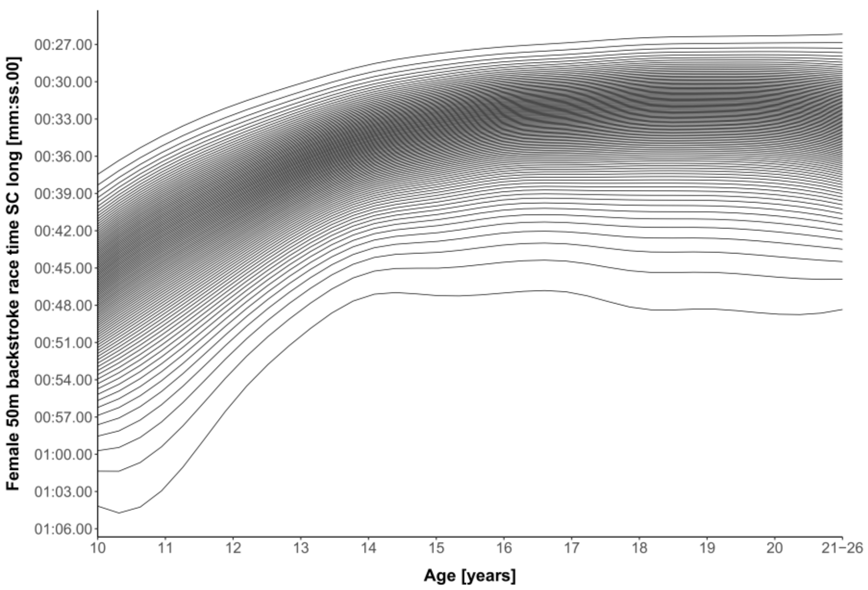

F_BA_100

Cross-sectional analysis

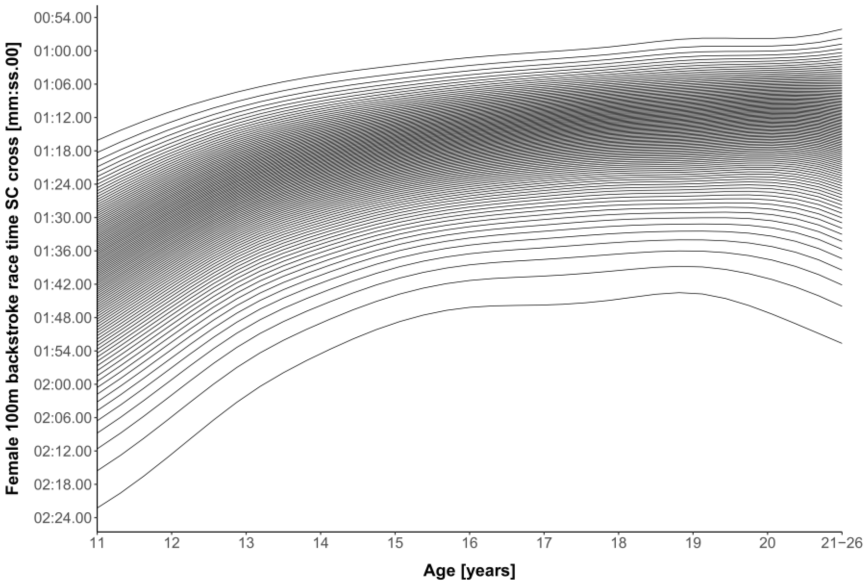

Longitudinal tracking

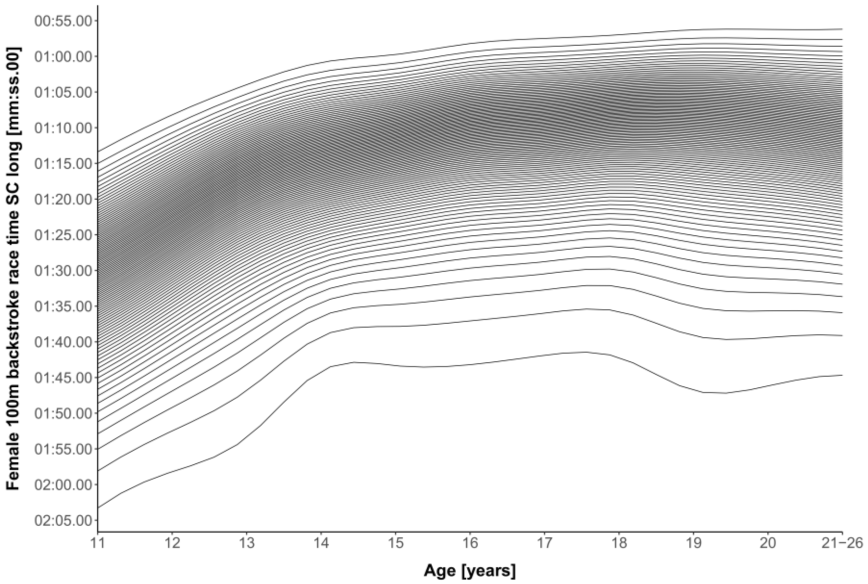

F_BA_200

Cross-sectional analysis	Longitudinal tracking
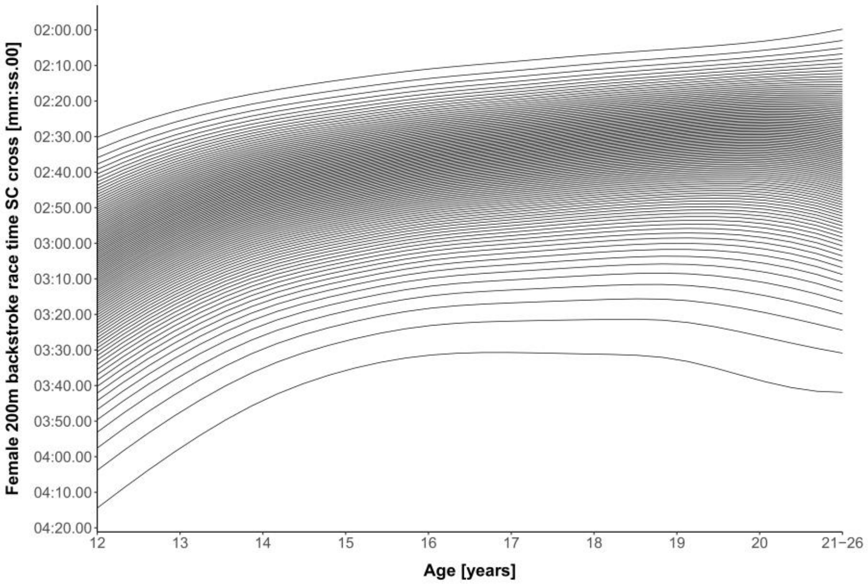	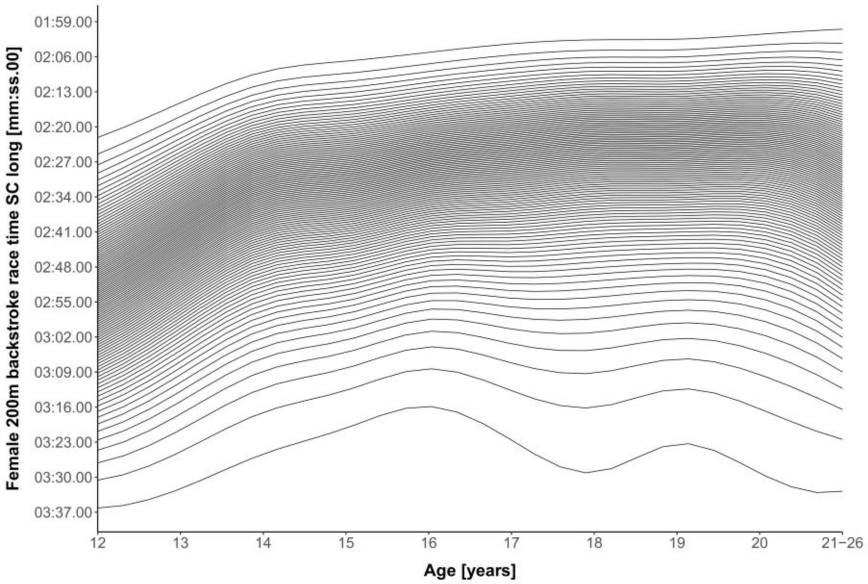

Breaststroke (BR)

F_BR_50

Cross-sectional analysis	Longitudinal tracking
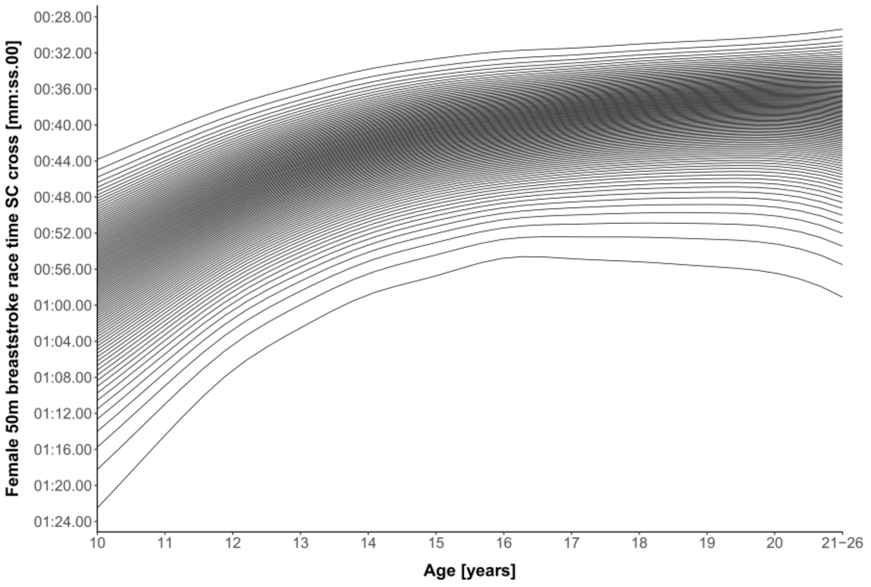	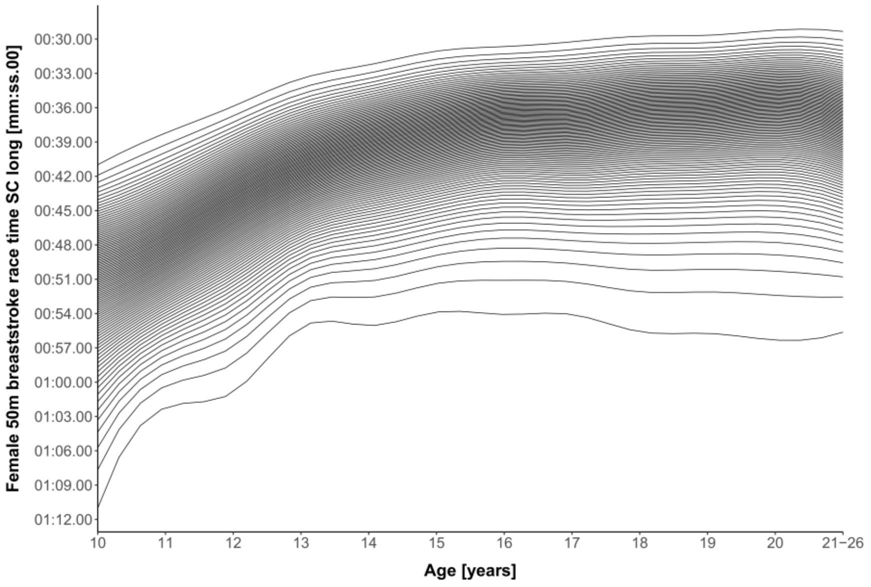

F_BR_100

Cross-sectional analysis

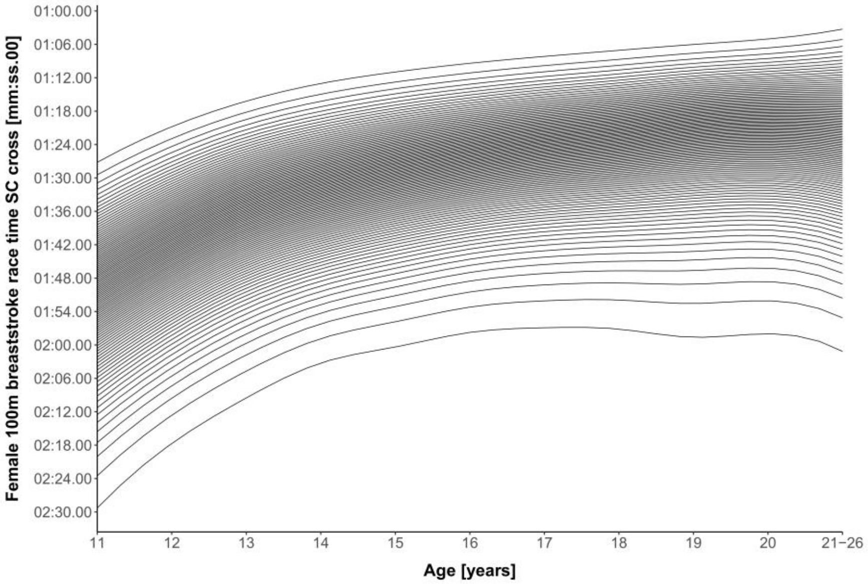

Longitudinal tracking

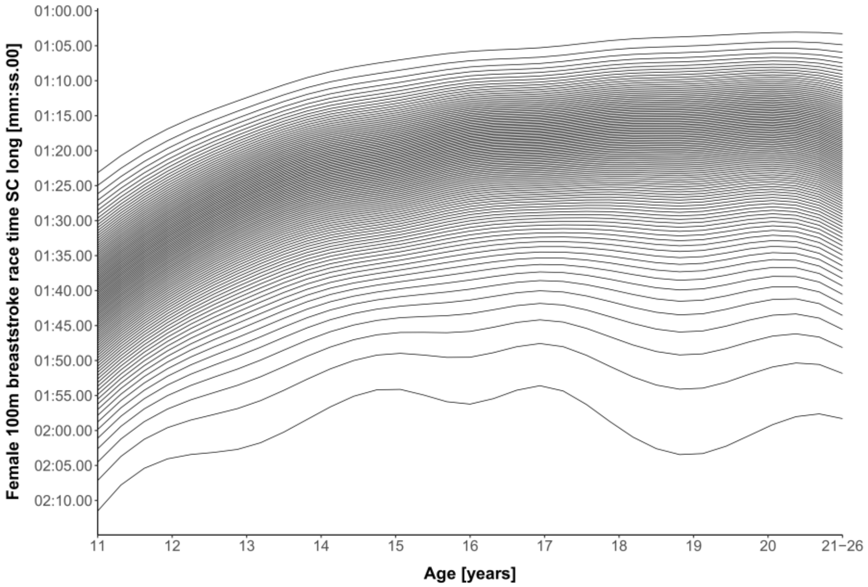

F_BR_200

Cross-sectional analysis	Longitudinal tracking
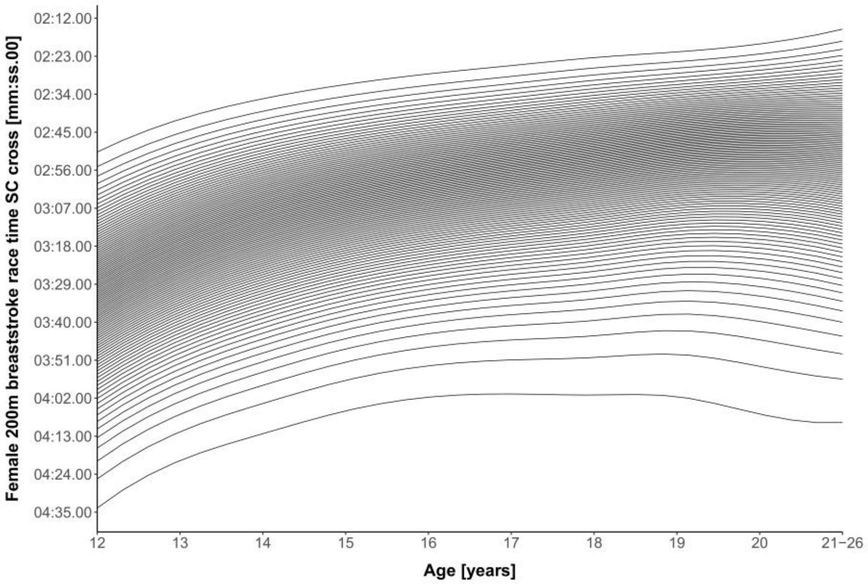	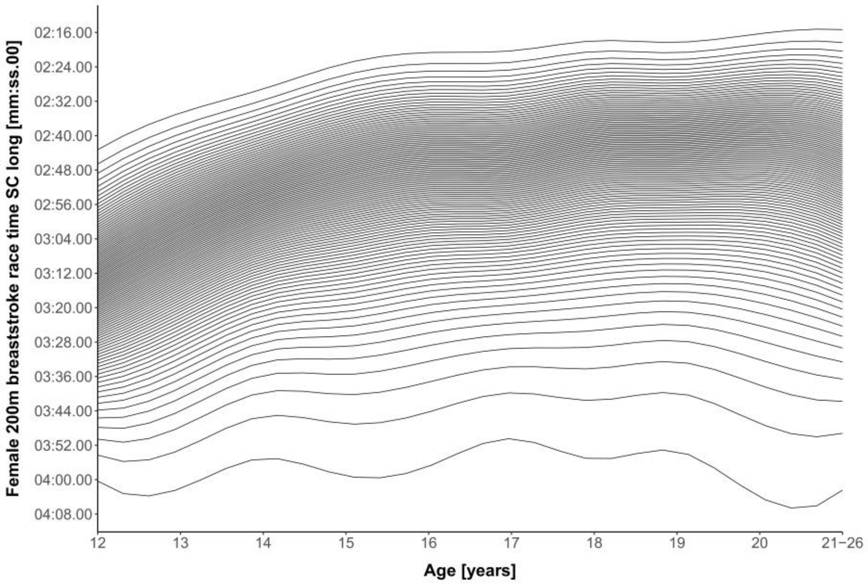

Freestyle (FR)

F_FR_50

Cross-sectional analysis	Longitudinal tracking
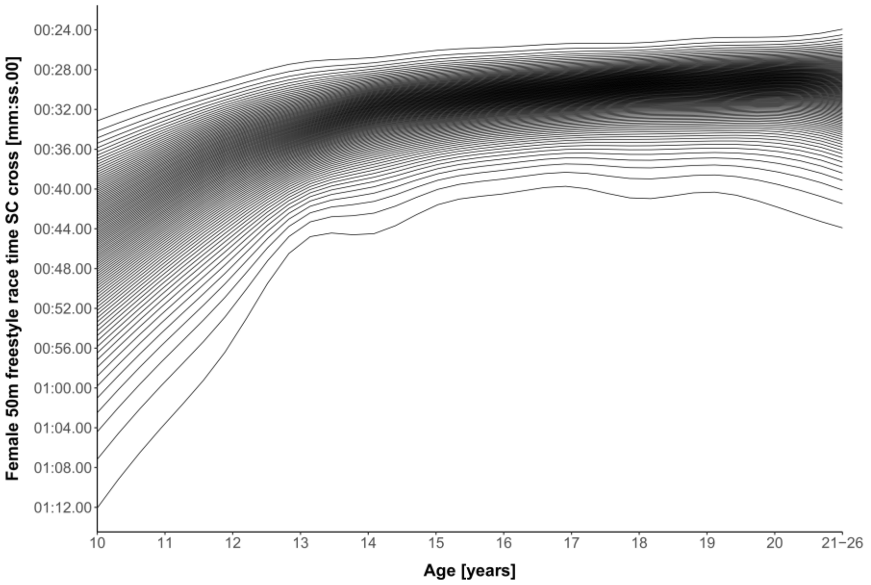	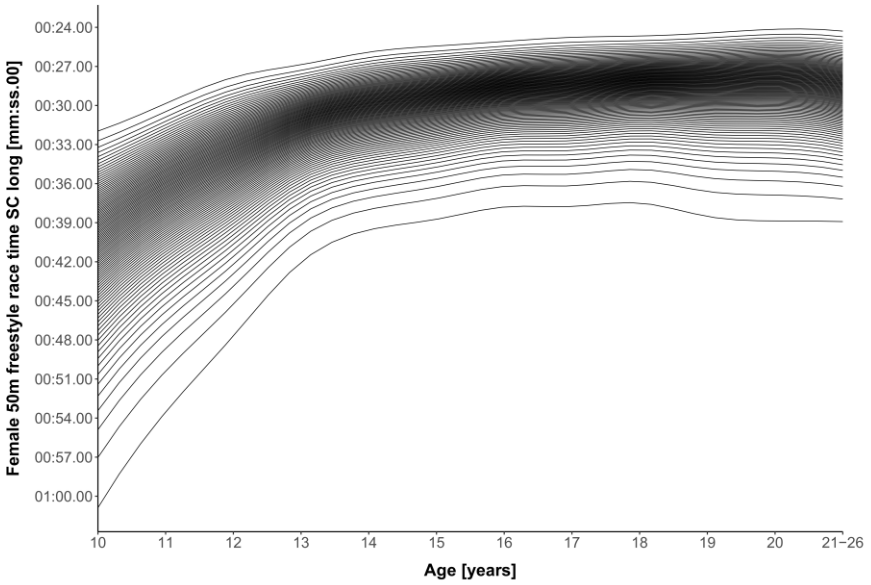

F_FR_100

Cross-sectional analysis	Longitudinal tracking
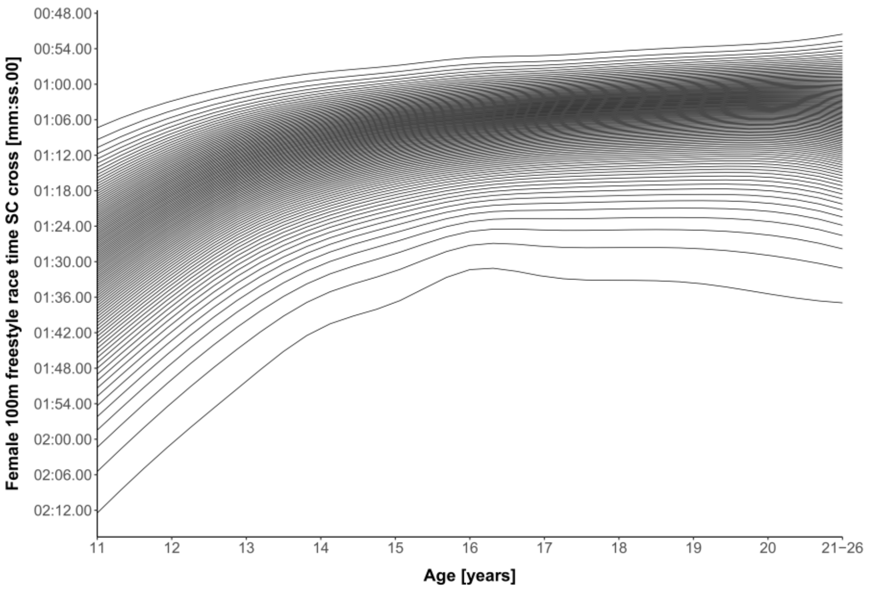	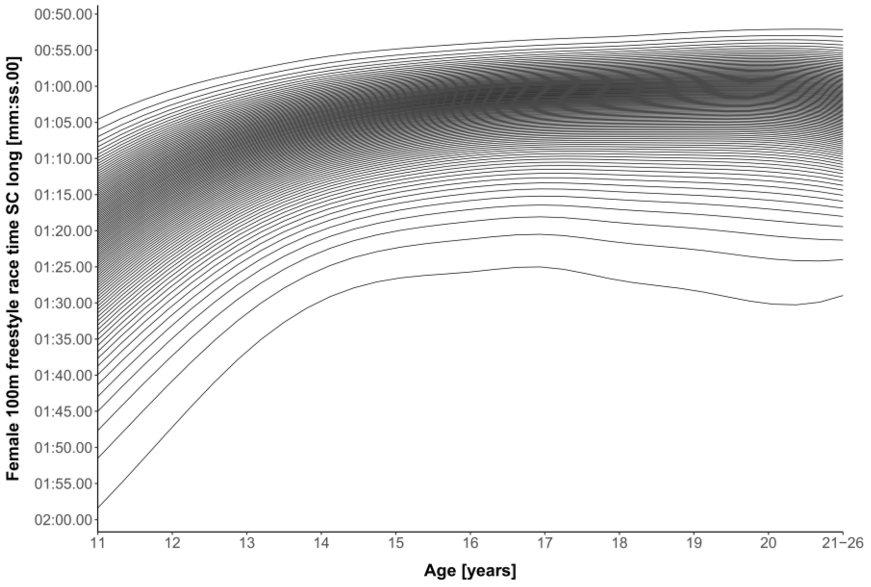

F_FR_200

Cross-sectional analysis

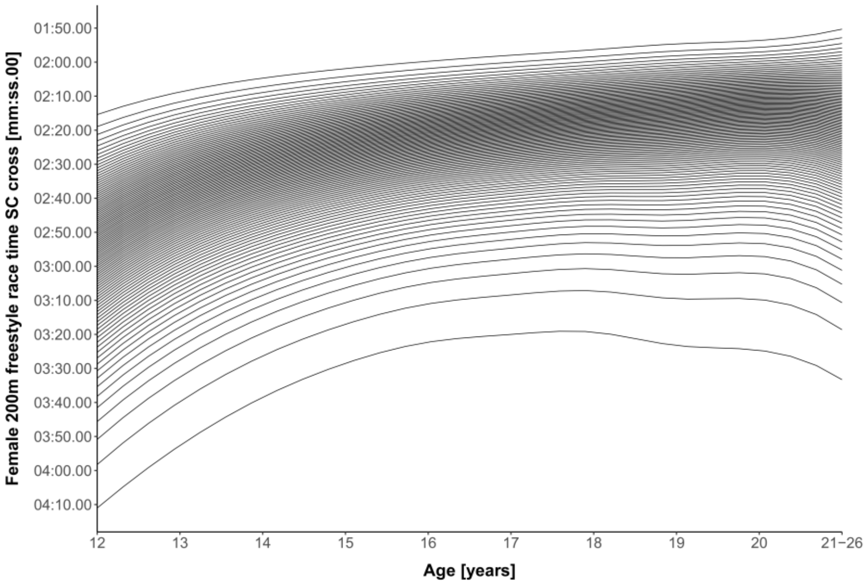

Longitudinal tracking

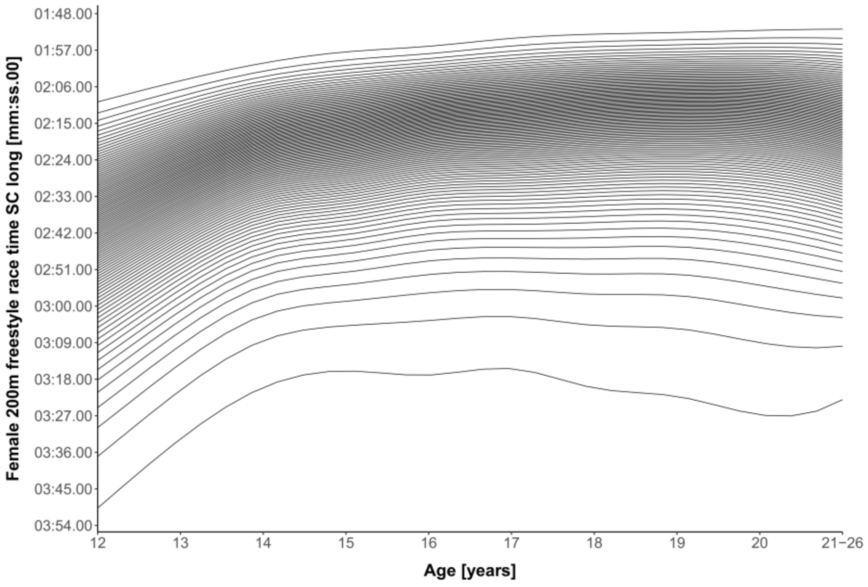

F_FR_400

**Cross-sectional analysis**	**Longitudinal tracking**
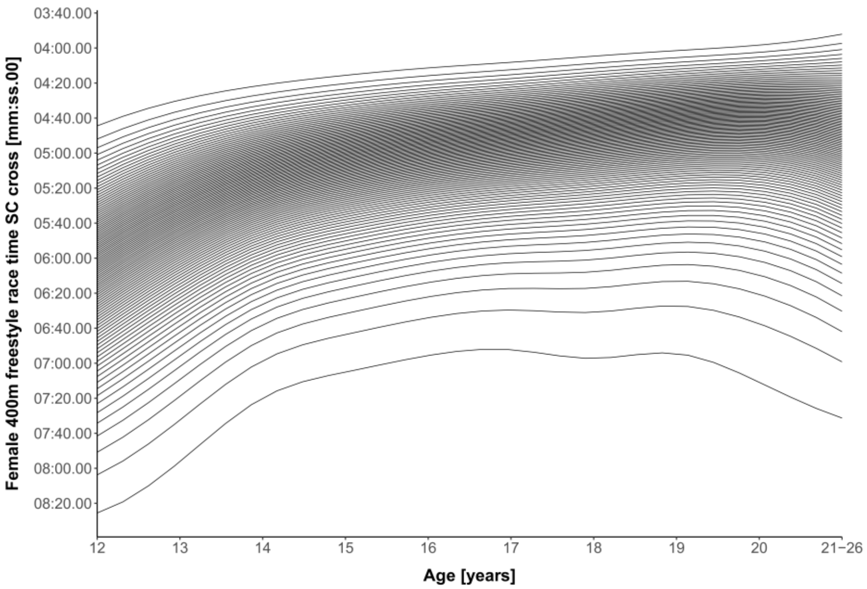	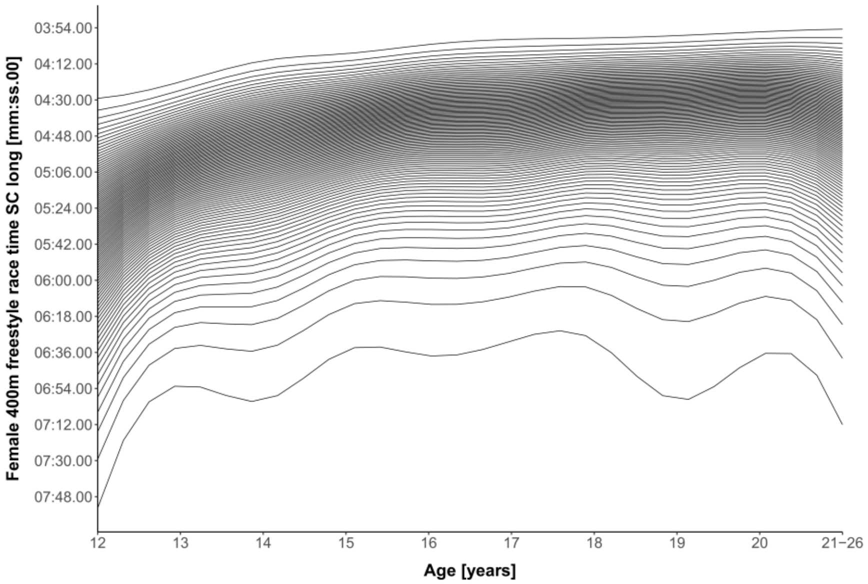

F_FR_800

Cross-sectional analysis

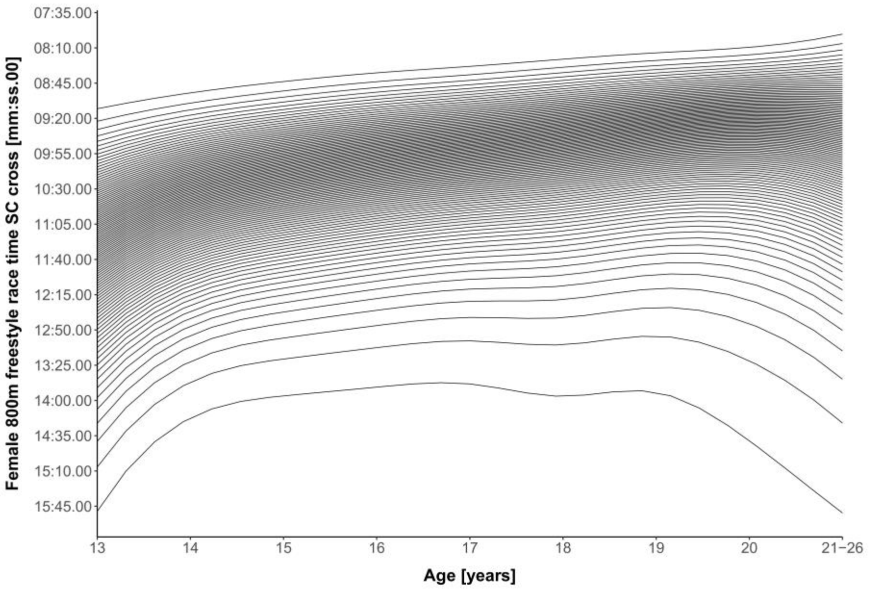

Longitudinal tracking

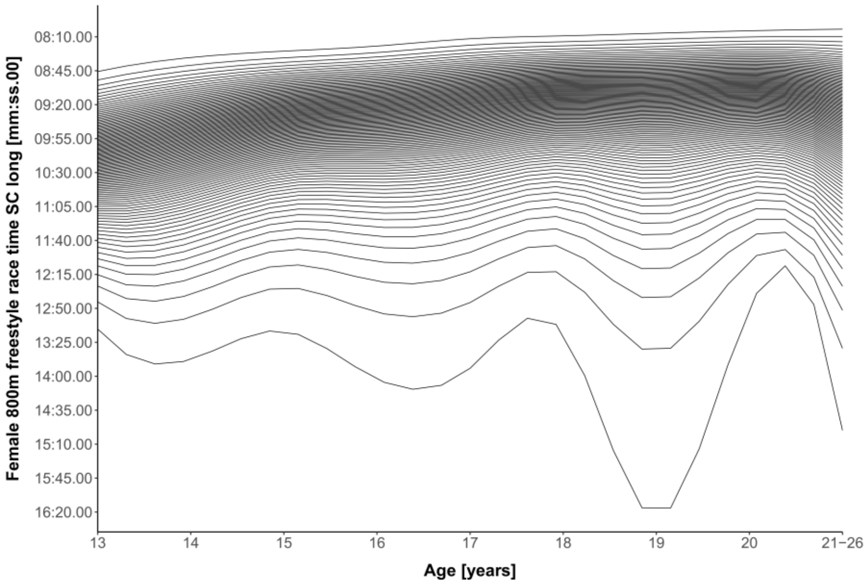

F_FR_1500

Cross-sectional analysis	Longitudinal tracking
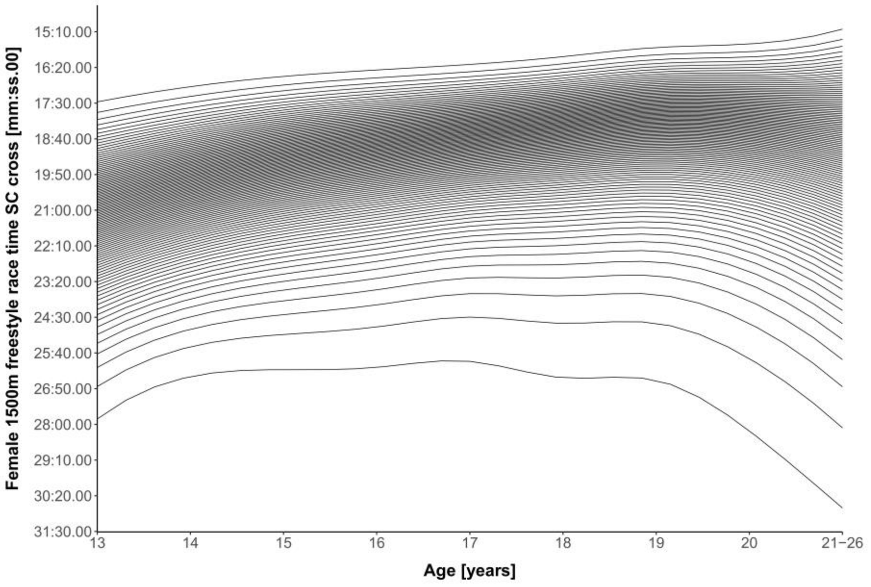	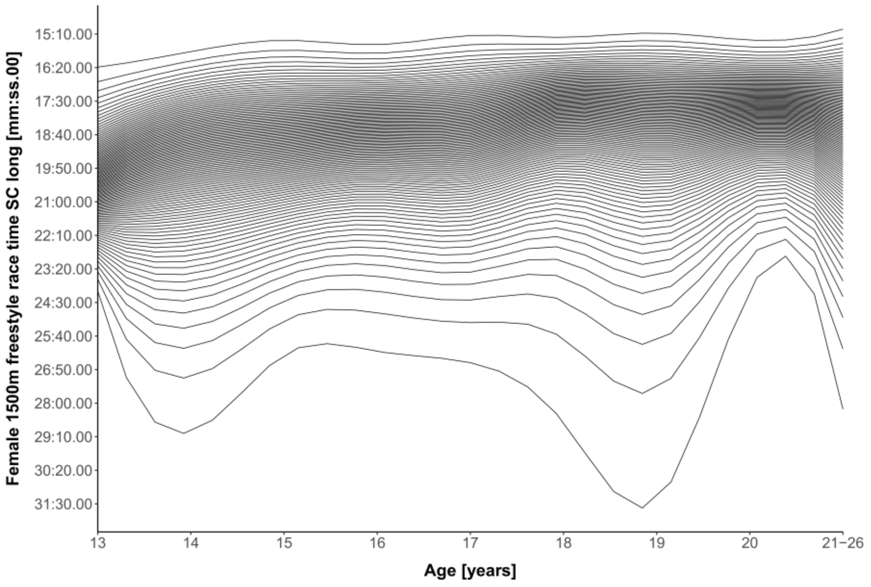

Individual medley (IM)

F_IM_200

Cross-sectional analysis	Longitudinal tracking
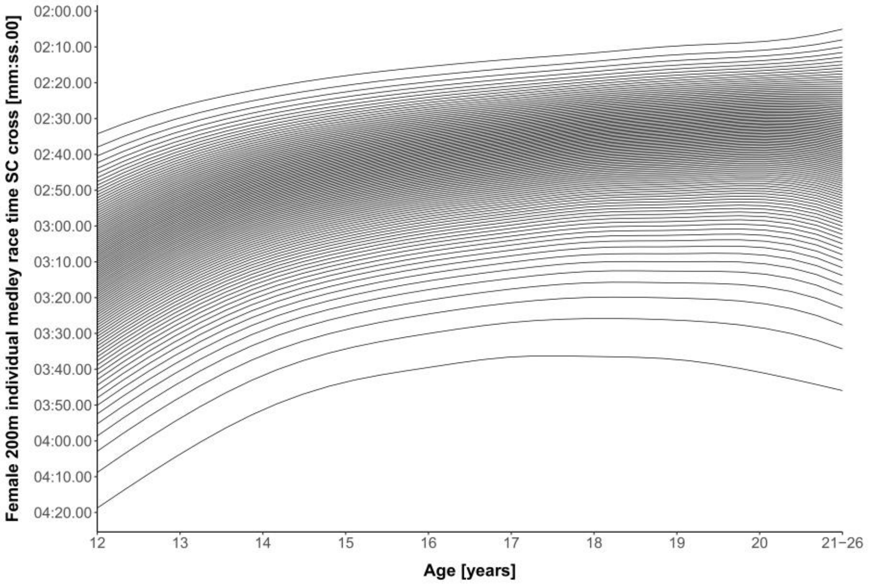	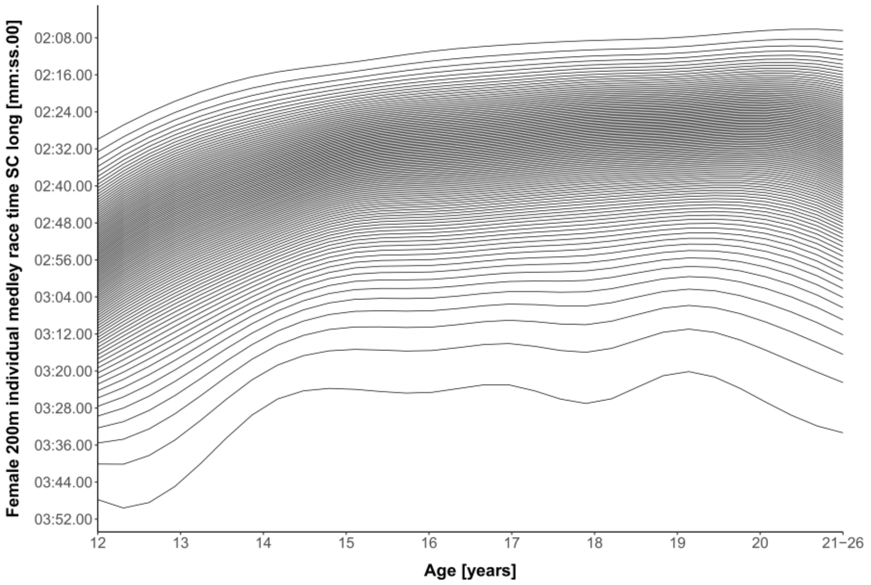

F_IM_400

Cross-sectional analysis

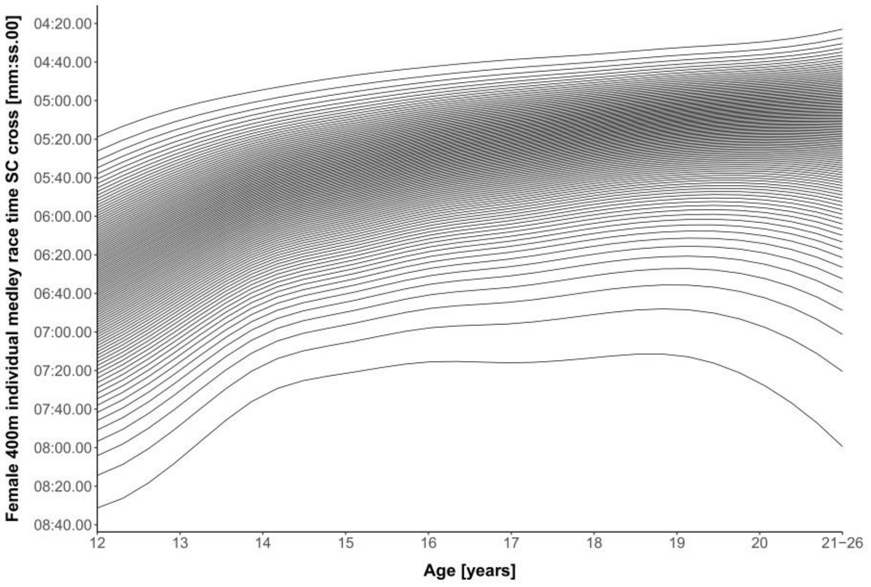

Longitudinal tracking

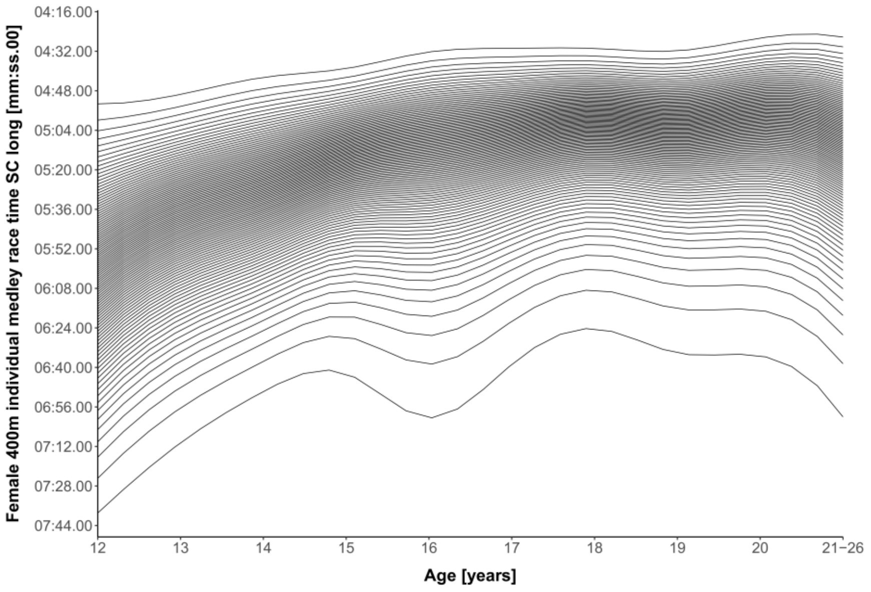

Supplement: Supplementary file 2 — Supplementary Information 2. [file 41598_2022_13837_MOESM2_ESM.pdf]
